# Novel genotype–phenotype correlations, differential cerebellar allele-specific methylation, and a common origin of the (ATTTC)_n_ insertion in spinocerebellar ataxia type 37

**DOI:** 10.1007/s00439-024-02644-7

**Published:** 2024-02-23

**Authors:** Marina Sanchez-Flores, Marc Corral-Juan, Esther Gasch-Navalón, Davide Cirillo, Ivelisse Sanchez, Antoni Matilla-Dueñas

**Affiliations:** 1 Neurogenetics Unit, Department of Neuroscience, Germans Trias i Pujol Research Institute (IGTP), Universitat Autònoma de Barcelona-Can Ruti Campus, Carretera de Can Ruti, Camí de les Escoles s/n, 08916 Badalona, Spain; 2 Barcelona Supercomputing Center (BSC), Barcelona, Spain

## Abstract

Spinocerebellar ataxia subtype 37 (SCA37) is a rare disease originally identified in ataxia patients from the Iberian Peninsula with a pure cerebellar syndrome. SCA37 patients carry a pathogenic intronic (ATTTC)n repeat insertion flanked by two polymorphic (ATTTT)n repeats in the Disabled-1 (DAB1) gene leading to cerebellar dysregulation. Herein, we determine the precise configuration of the pathogenic 5ʹ(ATTTT)n–(ATTTC)n–3ʹ(ATTTT)n SCA37 alleles by CRISPR–Cas9 and long-read nanopore sequencing, reveal their epigenomic signatures in SCA37 lymphocytes, fibroblasts, and cerebellar samples, and establish new molecular and clinical correlations. The 5ʹ(ATTTT)n–(ATTTC)n–3ʹ(ATTTT)n pathogenic allele configurations revealed repeat instability and differential methylation signatures. Disease age of onset negatively correlated with the (ATTTC)n, and positively correlated with the 3ʹ(ATTTT)n. Geographic origin and gender significantly correlated with age of onset. Furthermore, significant predictive regression models were obtained by machine learning for age of onset and disease evolution by considering gender, the (ATTTC)n, the 3ʹ(ATTTT)n, and seven CpG positions differentially methylated in SCA37 cerebellum. A common 964-kb genomic region spanning the (ATTTC)n insertion was identified in all SCA37 patients analysed from Portugal and Spain, evidencing a common origin of the SCA37 mutation in the Iberian Peninsula originating 859 years ago (95% CI 647–1378). In conclusion, we demonstrate an accurate determination of the size and configuration of the regulatory 5ʹ(ATTTT)n–(ATTTC)n–3ʹ(ATTTT)n repeat tract, avoiding PCR bias amplification using CRISPR/Cas9-enrichment and nanopore long-read sequencing, resulting relevant for accurate genetic diagnosis of SCA37. Moreover, we determine novel significant genotype–phenotype correlations in SCA37 and identify differential cerebellar allele-specific methylation signatures that may underlie DAB1 pathogenic dysregulation.

**Supplementary Information:**

The online version contains supplementary material available at 10.1007/s00439-024-02644-7.

## Background

Autosomal-dominant spinocerebellar ataxias (SCAs) are rare inherited movement neurodegenerative disorders mainly characterized by progressive cerebellar ataxia variably associated with ophthalmoplegia, pyramidal and extrapyramidal signs, dementia, pigmentary retinopathy, seizures, lower motor neuron signs, and peripheral neuropathy (Dueñas et al. 2006; Durr 2010; Klockgether et al. 2019). Disease onset is typically in adulthood, albeit some clinical signs can appear earlier. Currently, 48 well-defined dominant ataxia subtypes have been described evidencing the high clinical, genetic, and neuropathological heterogeneity. Genetic defects have been associated in 42 subtypes, playing a prominent role underlying SCAs physiopathology, with tandem repeat expansions (TREs) or conventional mutations triggering toxic gain- or loss-of-function events underlying neurodegeneration (Durr 2010; Jayadev and Bird 2013; Matilla-Dueñas et al. 2014; Tan et al. 2023). TREs, in both coding and non-coding genomic regions, represent one third of all the genetic defects associated with the SCAs and up to 60 monogenic disorders (Wen et al. 2023). They are highly polymorphic and reveal high instability in repeat length. Assessing their exact number is difficult by PCR amplification because of reduced amplification efficiency, allele dropout, and replication slippage defects limiting clinical-genetic correlations and genetic diagnosis (Polak et al. 2013; Hommelsheim et al. 2014; Potapov and Ong 2017; Kacher et al. 2021). Long pathogenic TREs are difficult to characterize by next-generation sequencing (NGS) based on short reads limited to fragments of 150 bp for their difficulty to be aligned to the reference genome leading to inaccurate sizing and misinterpretation.

We previously described the spinocerebellar ataxia subtype 37 (SCA37) in four unrelated Spanish kindreds caused by an unstable intronic (ATTTC)n pentanucleotide repeat insertion, located within an (ATTTT)n repeat tract in the 5’non-coding regulatory region of the *DAB1* gene encoding the Reelin adaptor protein, which is implicated in neuronal migration (Serrano-Munuera et al. 2013; Corral-Juan et al. 2018). The heterozygous (ATTTC)n repetitive insertion was identified in affected patients of all SCA37 families described to date from the Southeast of the Iberian Peninsula, four Spanish and six Portuguese, and, more recently, in a German kindred (Serrano-Munuera et al. 2013; Seixas et al. 2017; Corral-Juan et al. 2018; Rosenbohm et al. 2022). Normal non-pathogenic alleles show a repetitive ATTTT stretch of 7–400 units, which is not interrupted by ATTTC units, though 3% may be interrupted by AT-rich motifs; most (94%) bear ≤ 30 ATTTT repeats (Loureiro et al. 2019; Rosenbohm et al. 2022). Age-dependent penetrant pathogenic alleles include an insertion of 31–102 (ATTTC)n repeats flanked by adjacent ATTTT repeats larger than 58 units, 5ʹ(ATTTT)n–(ATTTC)n–3ʹ(ATTTT)n, being the 3’(ATTTT)n the largest described containing up to 420 repeat units (Rosenbohm et al. 2022), and without interruptions neither in the (ATTTT)n or the (ATTTC)n tracts (Loureiro et al. 2019). The ATTTC repeat inserted mutation dysregulates *DAB1* expression up-regulating Reelin-DAB1 and PI3K/AKT signalling in the SCA37 cerebellum (Corral-Juan et al. 2018).

Herein, we implement unbiased PCR-free amplification of targeted CRISPR/Cas9-mediated enrichment of the SCA37 altered region in combination with nanopore long-read sequencing to accurately determine the exact size and configuration of the 5ʹ(ATTTT)n–(ATTTC)n–3ʹ(ATTTT)n tract, obtain the methylation signatures of the SCA37 alleles, and generate predictive models for the age of onset and disease evolution using machine learning approaches. Moreover, we demonstrate a common origin of SCA37 chromosomes in the south of the Iberian Peninsula originating 859 years ago.

## Methods

### Patients and samples

DNA, genetic and clinical data of 24 individuals from four previously reported Spanish SCA37 unrelated kindreds (Serrano-Munuera et al. 2013; Corral-Juan et al. 2018), and 5 additional SCA37 individuals from four unrelated Spanish kindreds were included in this study. Informed consents were obtained for all individuals, and the study was approved by the clinical ethical board of the University Hospital Germans Trias i Pujol (HUGTP) in Badalona. Genetic and clinical data of 34 individuals from six previously reported Portuguese SCA37 kindreds (Seixas et al. 2017) were included to generate genotype–phenotype correlations and establish the origin and date of the SCA37 mutation in the Iberian Peninsula.

### Genetic and genomic studies

Genomic DNAs (gDNA) were isolated from peripheral blood leukocytes (PBL) from 29 SCA37 Spanish patients using Chemagen Magnetic Separation Module I automated system (Perkin Elmer). Additionally, high molecular weight (HMW) DNAs from one PBL, fibroblast cells (FC) from two patients and two cerebellar (CB) samples were extracted using the HMW DNA Extraction Kit, either for cells and blood (New England Biolabs (NEB), Cat. no. T3050S) or for tissue (NEB, Cat. No. T3060S). All 29 affected SCA37 members from eight Spanish SCA37 kindreds were genotyped for the non-pathogenic (ATTTT)n and the (ATTTC)n expanded insertion with a modified SCA37 long-PCR amplification protocol using LA Taq DNA polymerase (TaKaRa, Cat. no. RR002AG) and Sanger sequencing. Primers sequences (Suppl. Table 1) and PCR conditions are included in Supplementary Information.

### Unbiased long-read nanopore sequencing of the SCA37 region containing the (ATTTC)_n_ insertion

For nanopore sequencing of 11 SCA37 Spanish patients, the wild-type (ATTTT)n and SCA37 5’(ATTTT)n–(ATTTC)n–3ʹ(ATTTT)n genomic regions were targeted enriched by CRISPR–Cas9 to obtain high coverage using six crRNAs complementary to strands flanking the SCA37 5ʹ(ATTTT)n–(ATTTC)n–3’(ATTTT)n tract spanning a total of 22.29 kb within intron 11 of the *DAB1* gene (chr1: 57353671–57375963; hg38; Suppl. Figure 1a). Guides sequences and the detailed procedure of CRISPR–Cas9 assembled RNP complexes are described in Supplementary Table 2. DNA libraries were prepared according to previously published nCATS protocol (Gilpatrick et al. 2020) following the manufacturer’s protocol for SQK-LSK109 kit (Oxford Nanopore Technologies, ONT) with the following modifications. Briefly, up to 7 µg of gDNA and 120 units of thermolabile proteinase K (NEB, Cat. no. P8111S) were used to remove the Cas9 protein bound to DNA, which could interfere with adapters ligation, as recently suggested by Keraite and collaborators (Keraite et al. 2022). The library was eluted in 30 µL of elution buffer. The detailed protocol is described in Supplementary Information. DNA libraries were sequenced during 72 h in a PromethION sequencer (ONT), using a FLO-PRO002 flow cell (R9.4.1) per sample. Fast5 (electronic raw signal) and FASTQ (base called data) files were generated in real time with MinKNOWN software v22.08.6 and used for downstream bioinformatics analysis. Base calling modes Fast, Hac (high accuracy) and Sup (super high accuracy) from Guppy v 6.2.11 software were used to analyse the effect of these three different data conversions (fast5 to FASTQ) modes on sequence accuracy of the CRISPR–Cas9 targeted region. Sup mode requires a higher computing infrastructure for optimal base calling performance using Graphics processing unit (GPU). Raw signal was base called using the Sup mode in Guppy software v 6.2.11. All sequenced reads were aligned to the hg38 human reference genome with Minimap2 v2.17-r941 (Li 2018). SAMtools v1.10 (Danecek et al. 2021) was used for BAM files generation and Alfred v0.2.6 (Rausch et al. 2019) in combination with NanoStat v1.6.0 (De Coster et al. 2018) software was used for nanopore sequencing quality control and assessment of multiple-guide CRISPR–Cas9 enrichment. We calculated the genome-wide off-target depth of coverage per every 5000 bp using Mosdepth v0.3.4 in combination with the Cas-OFFinder tool and the RepeatMasker track (Tarailo‐Graovac and Chen 2009; Bae et al. 2014; Pedersen and Quinlan 2018). On-target sequencing reads were visualized with Integrative Genomics Viewer (IGV) v2.12.0 (Robinson et al. 2011). Since STRique, a Python package to analyse repeat tracts, was not designed to count complex repeats such as those in SCA37 (Erdmann et al. 2023), MarginPhase (Ebler et al. 2019) and WhatsHap (Martin et al. 2016) software for phasing genomic variants were used in combination with Python3-based scripts Repeat Analysis Tools (https://github.com/PacificBiosciences/apps-scripts/tree/master/RepeatAnalysisTools) to classify the reads aligned to the repeat tract region and determine their repeat tract configuration. The mean of the standard deviation from interquartile ranges previously reported in Rosenbohm and collaborators (Rosenbohm et al. 2022) was calculated with interquartile range (IQR) formula (SD = IQR/1.35) (Wan et al. 2014a). Absolute repeat instability index was calculated as previously described (Lee et al. 2010) considering a threshold of more than one read contiguously present in reads distribution. Modifications outlined by Nakamori (Nakamori et al. 2020) were applied to calculate relative instability index in the cerebellum or skin fibroblasts compared to peripheral blood lymphocytes from the same patient.

### Allele-specific methylation analysis and TF-binding sites enrichment

Haplotype reconstruction was used to assess allele-specific methylation with the f5c bioinformatic tool (Gamaarachchi et al. 2020). Methylation was predicted for individual CpG sites located in both strands of the target region. Outlier log-likelihood ratio (LLR) values were removed using the 1.5 × IQR method using R script (Yang et al. 2019). Allele-specific methylation frequencies were calculated dividing the number of methylated reads by unmethylated reads on a particular position and considering a methylated position when a LLR threshold was ≥ 2.0, as previously established (Liu et al. 2021). Differentially methylated regions (DMR) were considered when the absolute difference in the average overall methylation frequency was greater than 0.05 between WT and SCA37 alleles as previously described (Grant et al. 2022). The method “loess” in ggplot2 R-package was used for smoothed data representation of methylation fraction values with span parameter set to 0.1. Log-likelihood ratio values were also used to determine the significantly differentially methylated CpG sites in the SCA37 cerebellum with one-way ANOVA or the non-parametric Mann–Whitney *U* tests after Levene’s test of homogeneity of variances using an in-house R script. The transcription factor database JASPAR 2022 CORE vertebrate collection was used to predict TF-binding sites surrounding the CpG positions significantly differentially methylated in SCA37 cerebellum considering f5c LLR values and identified in the top five predictive models for age of onset and disease evolution (Castro-Mondragon et al. 2022). Disease evolution was considered as the time from the onset to the time when the sample was collected.

### Correlation analysis and linear regression models

All the following statistical analyses were performed using dedicated Python libraries. Two datasets were analysed. The first dataset consisted of 56 individuals from Spain and Portugal including clinical features and the repeat tract size and configuration. The second dataset consisted of nine SCA37 individuals from Spain who were sequenced by nanopore technology (eight blood samples, two skin fibroblasts samples, and two cerebellar samples). Nominal variables (gender, country/geographic area, tissue) were converted into integer labels, while numerical variables were standardized (*z*) considering mean (*μ*) and standard deviation (*σ*) as follows:

$$z = \frac{x - \mu }{\sigma }.$$

For regression analysis, ordinary least squares (OLS) linear regression models to examine the association between the dependent variable (“Age of onset”) and all possible pairs of independent variables were used. Models with coefficient *p *values of less than 0.05 were selected and ranked based on the coefficient of determination (*R*^2^) and the Bayesian Information Criterion (BIC). With *n* independent variables, the OLS regression model is

$$y = \beta_{0} + \sum\limits_{i = 1}^{n} {\beta_{i} x_{i} } + \in ,$$

where *y* is the dependent variable, *β*_0_ is the intercept of the model, *x*_*i*_ corresponds to the independent variable *i* of the model and ε is the random error. While the intercept represents the estimated value of the dependent variable when all independent variables are equal to zero, the coefficients (slopes) represent the change in the dependent variable for each unit change in the corresponding independent variable, holding the other variables constant.

Based on the first dataset containing data of Spanish and Portuguese patients, we generated models with the dependent variables “Age of onset” and “Disease evolution”, and the independent variables “Gender”, “WT-(ATTTT)n”, “WT total-repeat-length”, “SCA37-5’(ATTTT)n”, “(ATTTC)n”, “SCA37-3’(ATTTT)n”, “SCA37-total-repeat-length” and “Country”. Based on the second dataset containing data from blood samples sequenced by long reads, we generated models with the aforementioned dependent and independent variables in addition to “Age at sample collection” and “Differences in methylation frequencies” in 86 genomic regions containing 90 CpGs identified by the methylation algorithm. Subsets of these datasets with less than three data points or with any pair of independent variables with a strong Pearson’s correlation coefficient (*r* >  ± 0.8) were excluded from the model.

Appropriateness of the linear regression model was evaluated by examining the residuals versus the fitted values plot. Residuals are differences between the observed and predicted values of the dependent variable based on the estimated regression coefficients and represent the unexplained variation in the dependent variable that is not accounted for by the regression model. Regression modelling was performed using the statsmodels Python package.

It is important to note that SCA37 is a very rare disease and obtaining post-mortem samples are extremely difficult. Because of this limitation, we did not generate any predictive models from the data obtained from the cerebellar or fibroblasts samples, only from blood samples.

### Variable importance

We determined the relative importance of the independent variables of a given regression model using the SHAP (SHapley Additive exPlanations) method (Lundberg and Lee 2017). SHAP values can be computed for individual predictions or for the overall model, allowing for global and local interpretation. The importance of a variable was determined by calculating the proportion of the model's output variability that can be attributed to each feature in the complete dataset. SHAP values were calculated using the SHAP Python library.

### Estimation of the (ATTTC)_n_ SCA37 mutation age and haplotype reconstruction

To estimate the mutations’ age, 7 informative single-nucleotide polymorphisms (SNPs) located upstream and 17 located downstream of the (ATTTC)n SCA37 mutation, considering *DAB1* 5ʹ to 3ʹ direction, were used to reconstruct allele-specific haplotypes (Suppl. Figure 2). Primer sequences (Suppl. Table 1) and PCR and Sanger sequencing conditions for SNPs genotyping are included in Supplementary Information. The test for linkage disequilibrium was based on the Chi-square test.

BAM files from nanopore sequenced samples containing the on-target aligned long reads were processed with MarginPhase v1.0.0 (Ebler et al. 2019) for variant calling with a variant quality filter threshold > 30 and subsequently manually revised. WhatsHap v1.6 (Martin et al. 2016) was used for wild-type and SCA37 allele reads discrimination and haplotype reconstruction. A total of 30 SCA37 patients carrying the (ATTTC)n mutation from eight Spanish and six Portuguese unrelated kindreds were analysed. Whole-genome sequencing data previously generated from two SCA37 Spanish patients (Corral-Juan et al. 2018) were used to obtain the genotype information for 24 SNPs surrounding the (ATTTC)n mutation, spanning 6.8 Mb. Portuguese genotypes for SCA37 families (PO- M, G, R, MS, C, D) and controls were obtained from previous publication (Seixas et al. 2017). Imputation of missing genotypes and haplotype inference was performed using PHASE v2.1.1. (Stephens et al. 2001; Stephens and Donnelly 2003) with allele frequencies reported in Seixas et al*.* (Seixas et al. 2017) and NCBI SNP database (Sherry et al. 2001).The age of SCA37 (ATTTC)n mutation was estimated using DMLE+ v2.3 (Reeve and Rannala 2002). The relative position of the mutation was set within the haplotype considering 1 Mb = 1 cM according to physical distances given in the UCSC Genome Browser (Kent et al. 2002). Population growth rate of 0.167 was estimated based on population size, considering the oldest records from 1860 and the most recent ones for Spanish and Portuguese populations (htttp://www.ine.es/ and htttp://www.ine.pt), as reported by Russo et al. (2011). The proportion of chromosomes bearing the mutation was estimated considering a global SCA37 frequency in the Iberian Peninsula population of 106 affected chromosomes in a current population of 57959836 individuals (Slatkin and Rannala 1997). Phylogenetic networks were performed using 24 SNPs and generated with Network 10.2.0.0. (Bandelt et al. 1999) and POPTREE2 (Takezaki et al. 2010).

## Results

### Unbiased long-read nanopore sequencing of the (ATTTT/ATTTC)n repeat genomic region within the *DAB1* gene

We sequenced the (ATTTT/ATTTC)n repeat genomic region within the *DAB1* gene in 14 DNA samples including 10 PBL, 2 cerebellar (CB) and 2 fibroblasts (FC) samples obtained from 11 SCA37 patients of eight Spanish SCA37 kindreds. The mean age of patients at the time of sample collection was 57.15 years for PBLs (range: 47–75 years; SD ± 10.50), 72.50 for cerebellum (67 and 78 years; SD ± 7.78) and 62 for skin fibroblasts (50 and 74 years; SD ± 17). The mean age at onset was 43.67 years (range: 32–64; SD ± 9.77), while the mean of disease evolution was 18.9 years (range: 4–38; SD ± 11.2, the disease evolution for patient I:1 from AT-E was not available) (Suppl. Table 3). No information was available for I:1 and IV:9 patients from family AT-H and AT-9012, respectively. The unstable (ATTTC)_n_ pentanucleotide repeat inserted mutation located in the SCA37 genomic region within intron 11 of the *DAB1* gene was determined by long-PCR Sanger sequencing (Table 1) with (ATTTC)n ranging from 46 to 80 repeats (average = 58.42; SD ± 11.35) and a pure WT-(ATTTT)n allele ranging from 8 to 17 (average = 11.60; SD ± 3.22).

**Table 1:** Cas9-mediated long-read sequencing for DAB1 repeat region of Spanish wild-type and SCA37 chromosomes

				WT-(ATTTT)_n_			SCA37-5ʹ(ATTTT)n–(ATTTC)_n_–3ʹ(ATTTT)_n_							
Ped.	Ped. ID	Sample	Type of sample	WT-(ATTTT)_n_ repeats by sanger	Median WT-(ATTTT)_n_ repeats by nanopore	WT-(ATTTT)_n_ repeats by nanopore SD	(ATTTC)n repeats by sanger	Median (ATTTC)_n_ repeats by nanopore	(ATTTC)_n_ repeats by nanopore SD	5ʹ(ATTTT)_n_ repeats by nanopore	5ʹ(ATTTT)_n_ repeats by nanopore SD	3ʹ(ATTTT)_n_ repeats by nanopore	3ʹ(ATTTT)_n_ repeats by nanopore SD	Total n of reads (WT/SCA37)
AT-901	IV:9	SPA001	Blood	**9**	**9**	0.67	**48**	**48**	2.4	**66**	2.4	**83**	2.9	212 (113/99)
			Cerebellum*	**9**	**9**	0.66	**55**	**51**	3.5	**65**	2.6	**80**	5.6	482 (260/222)
	IV:10	SPA002	Blood	**9**	**9**	0.72	**46**	**48**	1.9	**66**	2.7	**83**	2.6	32 (16/16)
			Cerebellum*	**9**	**9**	0.65	**55**	**51**	3.2	**65**	2.8	**80**	4.3	441 (239/202)
	IV:4	SPA003	Fibroblasts*	**8**	**8**	0.59	**47**	**47**	2.9	**67**	2.7	**84**	3.1	644 (321/323)
AT-9012	III:3	SPB001	Blood	**8**	**8**	0.95	**51**	**50**	3.8	**67**	1.7	**85**	3.2	11 (7/4)
	IV:9	SPB002	Blood*	**11**	**11**	0.48	**58**	**59**	2.8	**69**	2.8	**84**	2.9	233 (131/102)
AT-59	IV:9	SPC001	Blood	**17**	**18**	1.18	**71**	**72**	1.7	**60**	2.2	**86**	5.4	39 (25/14)
			Fibroblasts*	**17**	**17**	1.11	**74**	**72**	4.5	**57**	2.7	**81**	3.4	907 (545/362)
AT-90	III:6	SPD001	Blood	**13**	**13**	1.21	**51**	**50**	3.3	**76**	3.6	**81**	4.9	50 (29/21)
AT-E	I:1	SPE001	Blood	**12**	**12**	0.58	**54**	**55**	2.6	**63**	1.2	**91**	2.1	28 (16/12)
AT-F	I:1	SPF001	Blood	**16**	**16**	0.62	**54**	**52**	2.5	**69**	1.9	**86**	3.4	58 (39/19)
AT-G	I:1	SPG001	Blood	**13**	**13**	0.58	**74**	**72**	3.0	**69**	3.0	**84**	2.5	17 (13/4)
AT-H	I:1	SPH001	Blood	**11**	**11**	1.05	**80**	**78**	1.5	**62**	1.4	**75**	3.2	72 (41/31)

Complete repeat lengths and conformations were accurately assessed by long-read nanopore sequencing in all DNA samples. The mean coverage depth was 185 × for the total CRISPR–Cas9 targeted genomic region and 216 × for the SCA37-5ʹ(ATTTT)n–(ATTTC)n–3ʹ(ATTTT)n repeat tract region. Wild-type alleles sequenced by long reads showed a pure WT-(ATTTT)n configuration with n ranging from 9 to 18 repeats (SD ± 0.5–1.2). Pathogenic SCA37 repeat tract consisted of 47–78 (ATTTC)n repeats (SD ± 1.5–4.5) flanked by 5ʹ—(ATTTT)n ranging from 57 to 76 repeats (SD ± 1.2–3.6) and the 3ʹ(ATTTT)n ranging from 75 to 91 repeats (SD ± 2.1–5.6). Repeat variability was significantly higher for the 3ʹ(ATTTT)n (SD ± 3.9; F_1,15_ = 24.236, *p *value < 0.0001), (ATTTC)n (SD ± 3.5; F_1,16_ = 24.478, *p *value < 0.0001) and the 5ʹ(ATTTT)n (SD ± 2.7, F_1,16_ = 24.478, *p value* < 0.0001) compared to the WT-(ATTTT)n repeat (SD ± 0.8). Significant correlation was observed between Sanger and Nanopore sequencing for the WT-(ATTTT)n repeat allele (Spearman’s r = 0.999, *p *value < 0.0001 (*n* = 14) (Suppl. Figure 7a) and for the (ATTTC)n repeat in the expanded allele (Spearman’s *r* = 0.956, *p *value < 0.0001 (*n* = 14) (Suppl. Figure 7b). Although extraction methods were not compared using the same samples, HMW DNA extraction (Sample*) performed better on average coverage than automatic Chemagen Magnetic Separation Module

*Ped*. Pedigree

**Fig. 1:**
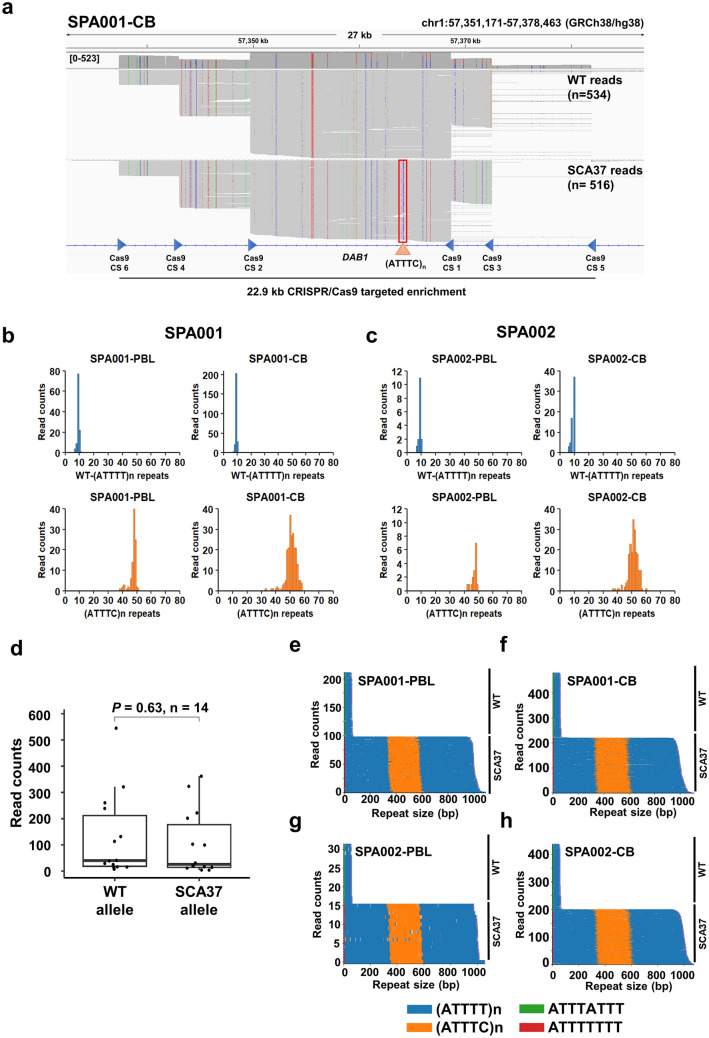
Long-read nanopore sequencing of the genomic region including the *DAB1* ATTTT/ATTTC repeat tract enriched by CRISPR**/**Cas9. **a** The integrative genomics viewer (IGV) showing the entire region of interest within *DAB1* intron 11 enriched by CRISPR/Cas9 successfully captured from SPA001 SCA37 patient’s cerebellum. Long sequenced reads were phased using WhatsHap for haplotype reconstruction for wild-type (top) and expanded (bottom) alleles. Read counts and repeat size for WT-(ATTTT)n and SCA37-(ATTTC)n from SPA001 **b** and SPA002 **c** blood lymphocytes and cerebellar samples. **d** No allele dropout was observed in read counts of expanded alleles compared to normal alleles (two sample *t *test; *p *value = 0.63, *n* = 14). Dot point indicates outlier WT read counts for the HMW extracted SPC0001 fibroblasts. Waterfall plots generated using Guppy Sup base calling mode showed pure (ATTTT)n and (ATTTC)n repeat tracts for SPA001 and SPA002 PBLs (**e** and **g**) and cerebella **f** and **h**. No interruptions were identified in any 5ʹ(ATTTT)n–(ATTTC)n–3ʹ(ATTTT)n repeats tract (Suppl. Figure 5). Relevantly, SCA37 alleles sequenced by long reads showed an ATTTTTTT sequence preceding the 5ʹ(ATTTT)n in SCA37 alleles in contrast to the ATTTATTT sequence preceding the WT-(ATTTT)n alleles

To determine the exact size and configurations of the 5'(ATTTT)n–(ATTTC)n–3'(ATTTT)n repeat tracts, we sequenced a 22.9 kb *DAB1* intronic region containing the repeat tract by enriching the targeted region with multiple-guide CRISPR–Cas9 and nanopore long-read sequencing (Fig. 1a). We evaluated enrichment efficiency by CRISPR–Cas9 using DNA samples from two healthy controls and four SCA37 patients previously genotyped for the *DAB1* repeat insertion. All guides presented a similar editing efficiency determined by qRT-PCR after 60 min of Cas9-mediated cleavage (mean cleavage efficiency = 85.8%; SD ± 4.2) (Suppl. Figure 1 and Suppl. Table 4). No significant differences were observed in Cas9-editing efficiencies between controls and SCA37 samples, suggesting that the DNA conformation arisen by the repetitive region did not interfere with Cas9-cleavage efficiency. The *DAB1* targeted loci was successfully captured with a mean coverage depth of 185 × for the CRISPR–Cas9 targeted genomic region and 216 × considering only the SCA37 repeat tract region (Suppl. Table 5). An enrichment of 521-fold above the expected background read coverage was achieved in the targeted region (2 × mean coverage depth for the whole genome; Suppl. Table 5). Genome-wide coverage analysis found the off-target reads to be distributed randomly across all chromosomes. Coverage analysis and manual inspection of off-target loci revealed that most of the regions did not encompass coding genes, but were located within centromeric, telomeric or repetitive regions, or in regions containing *in silico* predicted off-target Cas9 cuts as reported previously (Gilpatrick et al. 2020; Mizuguchi et al. 2021; Miyatake et al. 2022) (Suppl. Tables 4 and 6, Suppl. Figure 3). It is important to note that albeit the different DNA extraction methods were not compared in the same samples, high molecular weight DNAs overall performed better on sequencing coverage than samples extracted with standard automatic Chemagen Magnetic Separation (Suppl. Table 5 and Suppl. Figure 4).

### *DAB1* (ATTTT/ATTTC)n repeats size and configuration in SCA37

Since STRique, a Python package to analyse repeat tracts, was not designed to count complex repeats as in SCA37 (Erdmann et al. 2023), MarginPhase and WhatsHap softwares for phasing genomic variants were used in combination with RepeatAnalysisTool to determine copy numbers for the *DAB1* WT-(ATTTT)n and the pathogenic 5ʹ(ATTTT)n–(ATTTC)n–3ʹ(ATTTT)n repeat alleles (Table 1; Fig. 1b, c). No allele dropout was observed in read counts of SCA37 alleles compared to normal alleles (Fig. 1d; two sample *t* test; *P* = 0.63, *n* = 14). As expected, Sup base calling mode performed with better accuracy (96.1%) compared to Hac mode (95.7%; Suppl. Table 7). The average error rates per sample were 5.8% for fast mode (SD ± 0.9), 4.3% for Hac mode (SD ± 0.5) and 3.6% for Sup mode (SD ± 0.5) (Suppl. Table 7). Waterfall plots for each sample were generated showing the repeat size, configuration and composition using Sup base calling mode in combination with RepeatAnalysisTools (Fig. 1e–h; Suppl. Figure 5). Complete repeat lengths and conformations were accurately assessed with Sup base calling mode after comparing to Hac or fast modes (Suppl. Figure 6). Wild-type alleles had a pure WT-(ATTTT)n configuration with *n* ranging from 9 to 18 repeats (SD ± 0.5–1.2). The pathogenic SCA37 repeat tract consisted of 47–78 (ATTTC)n repeats (SD ± 1.5–4.5) flanked by 5ʹ(ATTTT)n ranging from 57 to 76 repeats (SD ± 1.2–3.6) and the 3ʹ(ATTTT)n ranging from 75 to 91 repeats (SD ± 2.1–5.6). Variability of repeat size number was higher for the 3ʹ(ATTTT)n (average of the SD ± 3.9; F_1,15_ = 24.236, *p *value < 0.0001), (ATTTC)n (average of the SD ± 3.5; F_1,16_ = 24.478, *p *value < 0.0001) and 5ʹ(ATTTT)n (average of the SD ± 2.7; F_1,16_ = 24.478, *p *value < 0.0001) all in SCA37 alleles compared to the WT-(ATTTT)n (average of the SD ± 0.8) in wild-type alleles (Table 1). By avoiding bias from PCR amplification, our strategy notably reduced repeat count variability compared with the mean standard deviations with PCR amplification and nanopore sequencing (ATTTC)n SD ± 21.86; 5ʹ(ATTTT)n SD ± 30.61 and 3ʹ(ATTTT)n SD ± 114.24) resulted in other studies (Rosenbohm et al. 2022).

Significant correlation was observed between Sanger and nanopore sequencing for the WT-(ATTTT)n repeat allele (Spearman’s *r *= 0.999, *p* value < 0.0001 (*n* = 14); Suppl. Figure 7a) and for the (ATTTC)n repeat in the expanded allele (Spearman’s *r* = 0.956, *p *value < 0.0001 (*n* = 14); Suppl. Figure 7b). No interruptions were identified in SCA37 pathogenic alleles in neither 5’(ATTTT)n, (ATTTC)n, or 3ʹ(ATTTT)n tracts, presenting all pure repeat tracts (Fig. 1d, e; Suppl. Figure 5). Remarkably, SCA37 alleles showed an –ATTTTTTT– sequence preceding the 5ʹ(ATTTT)n of the repeat tract. This is in contrasts with the –-ATTTATTT– sequence preceding the WT-(ATTTT)n allele (Fig. 1e–h; Suppl. Figures 5, 8). CRISPR–Cas9 targeted cleavage and long-read sequencing were able to effectively enrich the *DAB1* 5ʹ(ATTTT)n–(ATTTC)n–3ʹ(ATTTT)n repeat tract and accurately determine the repeat size and structure in all sequenced samples.

### *DAB1* (ATTTT/ATTTC)n tissue-specific instability in SCA37

**Fig. 2:**
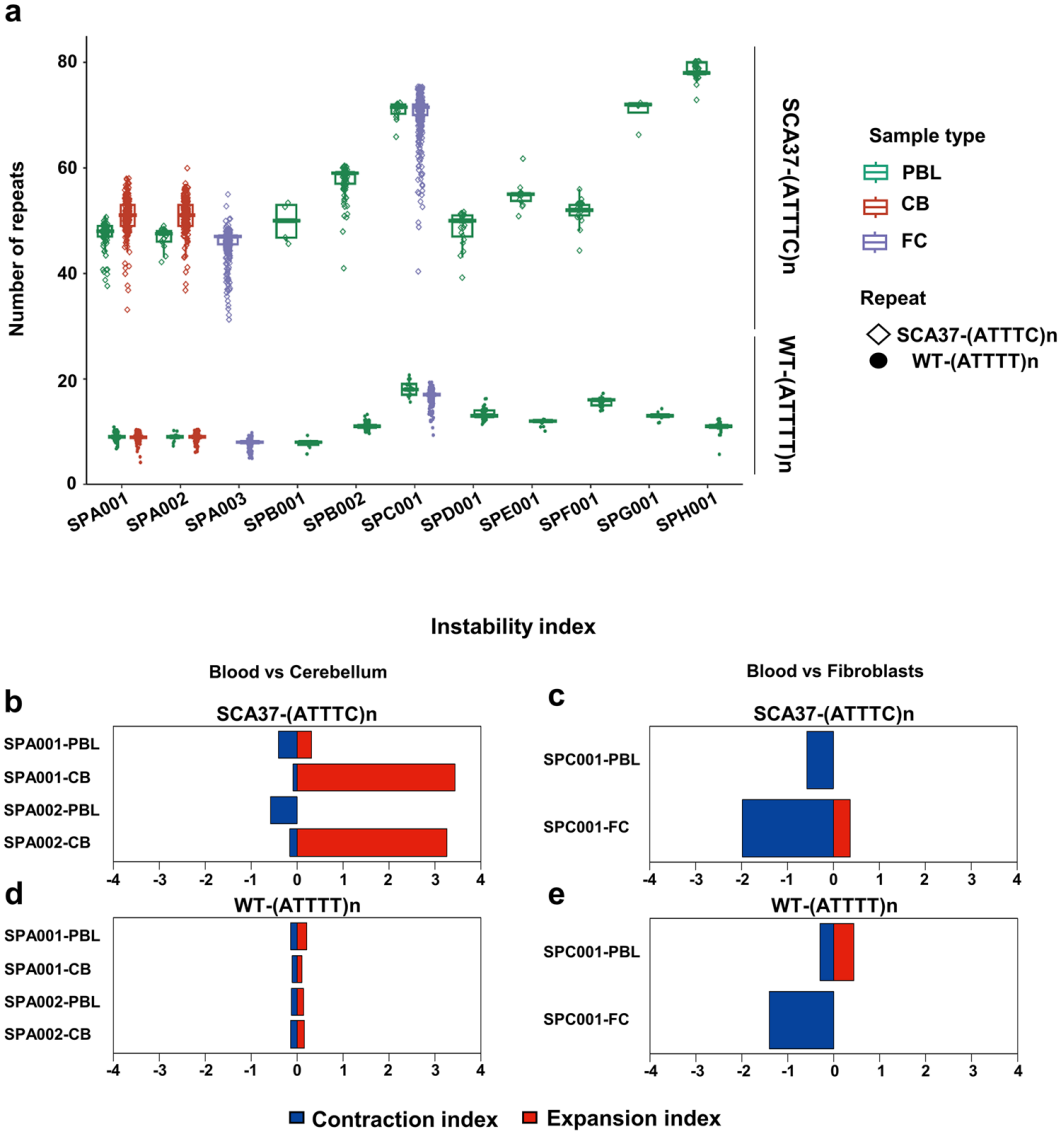
Tissue-specific length, variability, and instability index for the WT-(ATTTT)n and the inserted SCA37-(ATTTC)n repeated tracts. **a** Higher repeat variability was observed for the SCA37-(ATTTC)n repeat compared to the WT-(ATTTT)n. Slightly higher instability index biased towards contraction was observed for the SCA37-(ATTTC)n repeat compared to the WT-(ATTTT)n in blood samples **b**, **d**. **b** The (ATTTC)n instability index for cerebellum (average of instability index =  + 3.21) revealed an expansion-biased tissue-specific compared to blood samples (average of instability index =− 0.33). In fibroblasts, the instability index showed contraction for both the pathogenic (ATTTC)n **c** (instability index = 1.62) and the WT-(ATTTT)n (instability index =− 1.4) repeat tract **e**, compared to blood (average of instability index =− 0.33 for (ATTTC)n; average of instability index =  + 0.02 for WT-(ATTT)n; Suppl. Table 8 and Suppl. Figure 10)

We quantified the somatic instability index for the 5’(ATTTT)n, (ATTTC)n and 3’(ATTTT)n repeat tracts in cerebellum and fibroblasts cells relative to blood cells. Higher repeat variability was observed for the (ATTTC)n repeat (average of the SD ± 3.5) compared to the WT-(ATTTT)n (average of the SD ± 0.8) (Fig. 2a; Table 1). Slightly higher instability index biased towards contraction was observed for the (ATTTC)n repeat (average of instability index =—0.33) compared with the WT-(ATTTT)n (average of instability index =  + 0.02) in blood cells (Fig. 2b, d; Suppl. Figure 9 and 10,Suppl. Table 8). In addition, the (ATTTC)n instability index identified in cerebellum (average of instability index =  + 3.21) revealed an expansion-biased tissue-specific compared to blood cells (average of instability index =− 0.33) (Fig. 2b). In fibroblasts the instability index showed contraction for both (ATTTC)n (instability index = 1.62) (Fig. 2c) and WT-(ATTTT)n repeats (instability index =− 1.4) (Fig. 2e) compared to blood cells (average of instability index =− 0.33 for (ATTTC)n; average of instability index =  + 0.02 for WT-(ATTT)n) (Suppl. Figure 9 and 10).

**Fig. 3:**
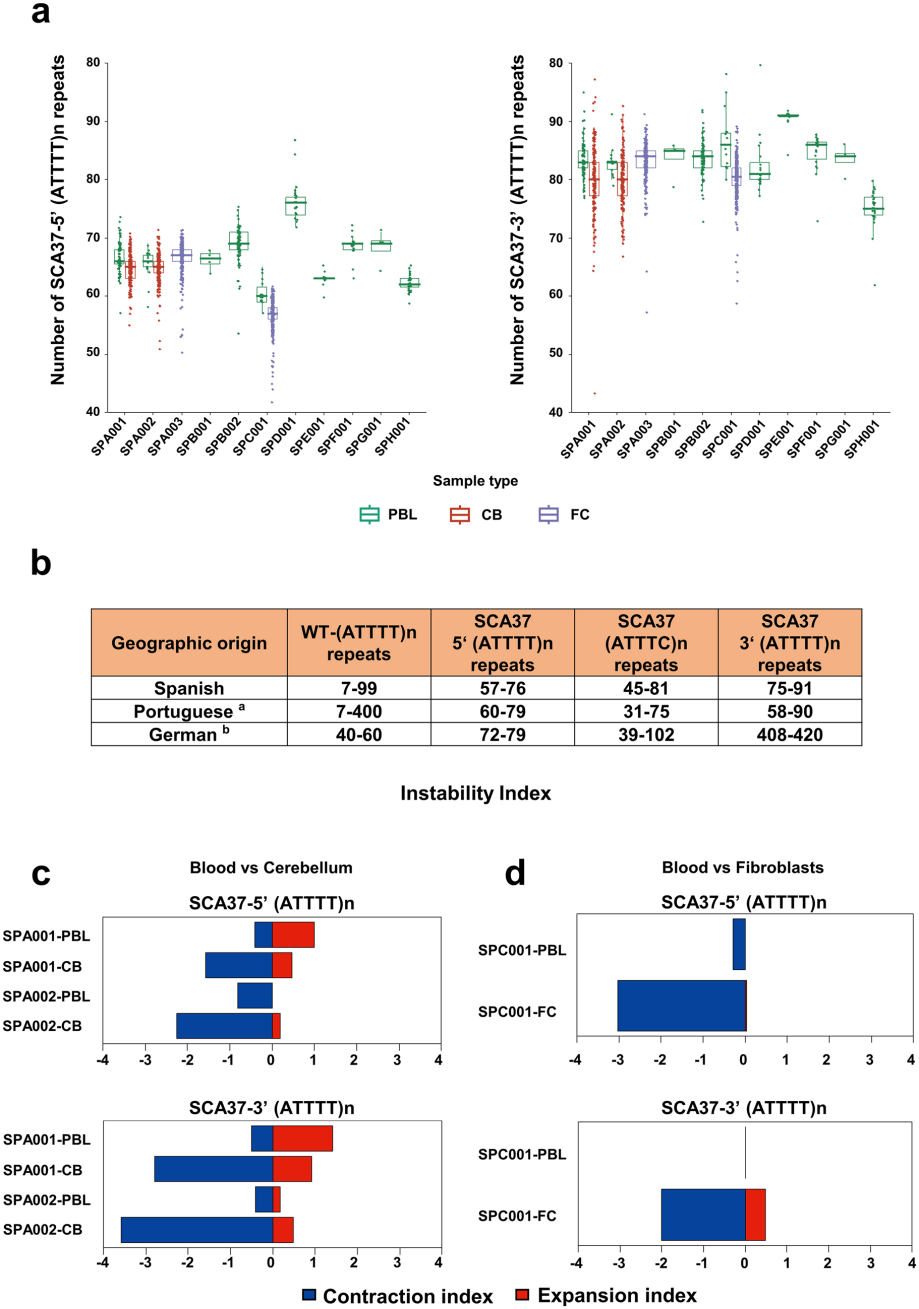
Repeat instability of the 5ʹ(ATTTT)n upstream and 3ʹ(ATTTT)n downstream of the inserted (ATTTC)n repeat in the mutant pathogenic allele. **a** The 3ʹ(ATTTT)n located downstream of the (ATTTC)n pentanucleotide repeat insertion (right) showed higher repeat variability (average SD ± 3.9) compared to the upstream 5ʹ(ATTTT)n (average SD ± 2.7) (left) in the SCA37 allele. **b** Remarkably, the 3ʹ(ATTTT)n presented the highest repeat variability between Spanish (75–91), Portuguese (58–90), and German (408–420) cases. **c** The instability index in both the 5ʹ(ATTTT)n and 3ʹ(ATTTT)n flanking the (ATTTC)n in the pathogenic alleles in cerebellum revealed a contraction-biased tissue-specific compared to blood samples (cerebellar 5’(ATTTT)n instability index =− 1.59; blood 5ʹ(ATTTT)n instability index = 0.11; cerebellar 3’(ATTTT)n instability index =− 2.5; blood 3ʹ(ATTTT)n instability index =  + 0.33). **d** In fibroblasts, the instability index also showed contraction biased for both 5ʹ(ATTTT)n and 3ʹ(ATTTT)n repeated tracts in pathogenic alleles compared to blood (fibroblasts 5’(ATTTT)n instability index =− 3.07; fibroblasts 3ʹ—(ATTTT) instability index =− 1.53) (Suppl. Table 8 and Suppl. Figure 10)

Furthermore, the 3'(ATTTT)n located downstream of the (ATTTC)n pentanucleotide repeat insertion showed higher repeat variability (average of SD ± 3.9) compared to the 5ʹ(ATTTT)n upstream variability (Average of SD ± 2.7) (Fig. 3a; Table 1). Remarkably, the 3’(ATTTT)n presented higher variability compared to the 5ʹ(ATTTT)n, within and between Spanish, Portuguese, and German patients (Fig. 3b) (Seixas et al. 2017; Corral-Juan et al. 2018; Loureiro et al. 2019; Rosenbohm et al. 2022). Both the 5ʹ(ATTTT)n and 3ʹ(ATTTT)n instability indices in cerebellum revealed a contraction-biased tissue-specific compared to blood cells, being higher in 3ʹ(ATTTT)n (cerebellar 5ʹ(ATTTT)n instability index =− 1.59; blood 5’(ATTTT)n instability index = 0.11; cerebellar 3’(ATTTT)n instability index =− 2.5; blood 3’(ATTTT)n instability index =  + 0.33) (Fig. 3c). Likewise, fibroblasts showed instability index towards contraction biased for both 5’(ATTTT)n and 3ʹ(ATTTT)n repeats compared to blood cells (fibroblasts 5ʹ(ATTTT)n instability index =− 3.07; fibroblasts 3ʹ(ATTTT) instability index =− 1.53) (Fig. 3d; Suppl. Figure 9 and 10, Suppl. Table 8). These data evidence the sizing and configuration accuracy of the new implemented method, the absence of interruptions in SCA37 pathogenic alleles and an expansion bias of the (ATTTC)n repeat and contraction bias of the 5’(ATTTT)n and 3’(ATTTT)n in SCA37 cerebellum relative to blood.

### Differential *DAB1* methylation signature in the SCA37 cerebellum

**Fig. 4:**
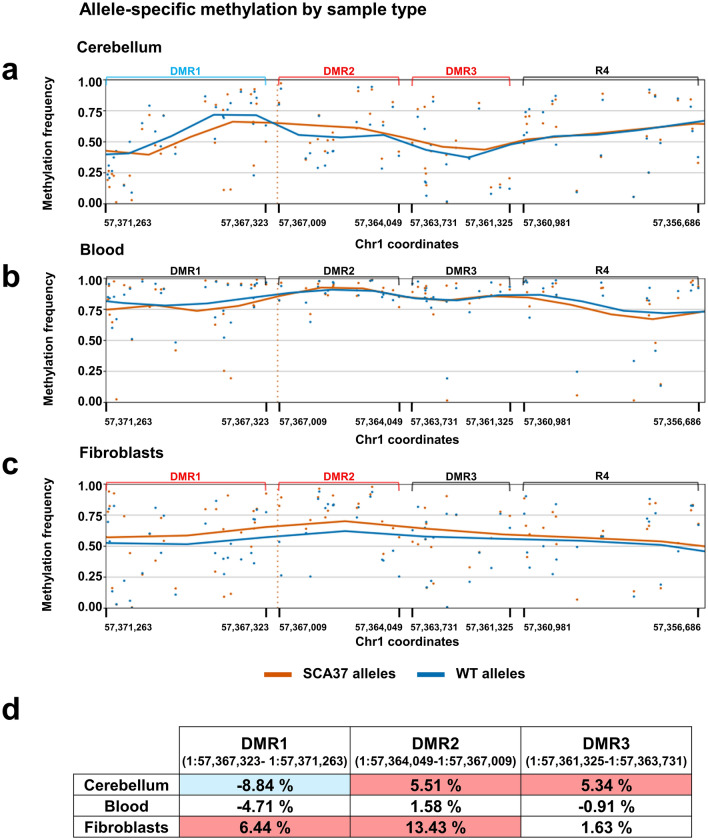
CpG methylation signatures of the SCA37 region within *DAB1* on 1p32 in cerebellum, peripheral blood cells and fibroblasts. For methylation studies, SCA37 and wild-type alleles were classified using WhatsHap software. **a** Similar allele-specific methylation signatures were present in two SCA37 cerebellar samples (SPA001-CB and SPA002-CB) compared to blood **b** and fibroblasts **c**. **d** Three global differentially methylated regions (DMR) were identified in SCA37 pathogenic alleles compared to WT-(ATTTT)n alleles. An hypomethylated region (R1) upstream of the 5’(ATTTT)n–(ATTTC)n–3ʹ(ATTTT)n SCA37 tract showed a 8.84% mean reduction of methylation frequencies ranging from “chr1:57367323” to “chr1:57371263”. In contrast, two regions, R2 and R3 downstream of the SCA37 repetitive tract, were found differentially hypermethylated compared to WT alleles, increasing 5.51% (R2; ranging from “chr1:57364049” to “chr1:57367009”) and 5.34% (R3; ranging from “chr1:57361325” to “chr1:57363731”) their global methylation frequencies. Blood samples did not reveal significant differences in methylation frequencies between WT and SCA37 alleles **b**. **c** Fibroblasts showed a slightly global increase of methylation frequencies in the SCA37 alleles compared to WT alleles with an increase of 6.44% in R1 (ranging from “chr1:57361325” to “chr1:57363731”) and 13.44% in R2 (ranging from “chr1:57364049” to “chr1:57367009”). The y-axis represents methylation frequencies shown in percentage and the genomic positions represented on the x-axis indicates DMR coordinates. Blue and orange lines represent smoothed methylation frequencies for wild-type and SCA37 disease alleles, respectively. The position of the 5ʹ(ATTTT)n–(ATTTC)n–3ʹ(ATTTT)n SCA37 tract is represented with a red vertical dotted line

A total of 55 informative SNPs within the on-target region were identified by nanopore sequencing and used to discriminate between wild-type or SCA37 allele reads within the on-target region (Suppl. Table 9). Differential CpG methylation frequencies (DMF) between wild-type and SCA37 alleles were quantified for blood cells (*n* = 10), fibroblasts (*n* = 2) and cerebellum (*n* = 2). Methylation analysis with the f5c software identified 86 potential methylated regions including 90 CpGs within the on-target region and 30 differentially methylated CpGs between SCA37 and wild-type cerebellar alleles (*p* value < 0.05), with 12 of them locating upstream and 18 downstream of the 5ʹ(ATTTT)n–(ATTTC)n–3’(ATTTT)n repeat tract (Suppl. Table 10). In cerebellum, three distinctive differentially methylated regions (DMR) adjacent to the 5’(ATTTT)n–(ATTTC)n–3’(ATTTT)n repeat tract were identified (Fig. 4a, d): one upstream hypomethylated region (DMR1 = − 8.84% ranging from chr1:57367323 to chr1:57371263) and two downstream hypermethylated regions (DMR2 = 5.51% ranging from chr1:57364049 to chr1:57367009; and DMR3 = 5.34% ranging from chr1:57,361,325 to chr1:57,363,731).

No relevant DMF (> 5%) between wild-type and SCA37 alleles were observed in blood in the same genomic regions (Fig. 4b, d). Fibroblasts presented a DMF of 6.44% for DMR1 (hypermethylation), a DMF of 13.43% for DMR2 (hypermethylation) and a DMF of 1.63% for DMR3, evidencing overall tissue-specific methylation (Fig. 4c, d) (Battaglia et al. 2022). Based on this evidence, we propose that the cerebellar-specific methylation signature identified in SCA37 alleles in this study may underlie DAB1 dysregulation shown in cerebellum of SCA37 patients (Corral-Juan et al. 2018). However, extending the methylation study including additional SCA37 alleles from control individuals and SCA37 patients would confirm the observed tissue-specific methylation effects identified.

### Novel SCA37 genotype–phenotype correlations

**Fig. 5:**
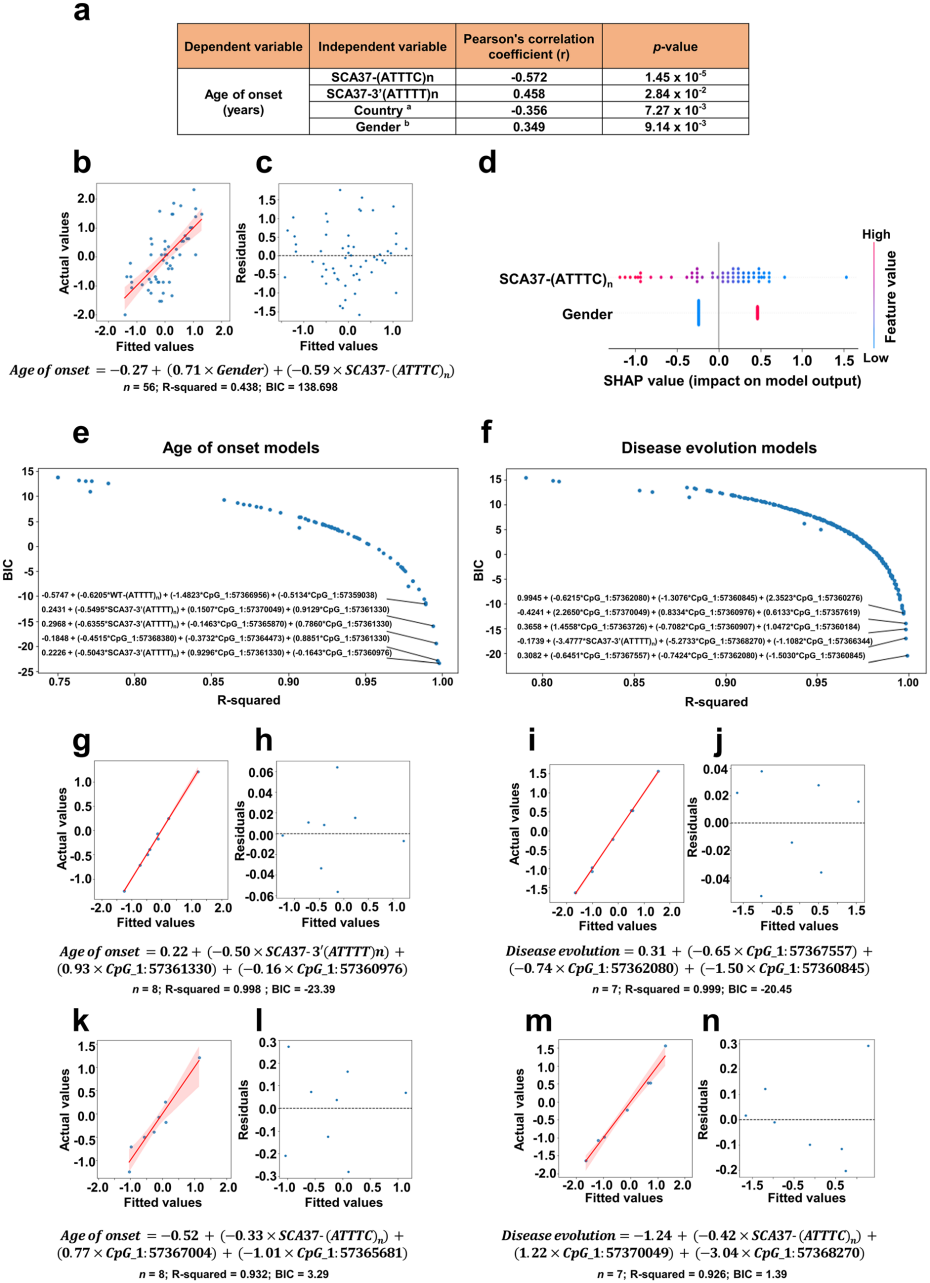
Novel genotype–phenotype associations and predictive linear regression models established in SCA37.** a** Significant Pearson correlation coefficients were obtained using “Age of onset” as dependent variable and the “SCA37-(ATTTC)n” (*n* = 56), “SCA37-3ʹ(ATTTT)n” (*n* = 22), “Country” (n = 56) and “Gender” (*n* = 56) as independent variables. **b** and** c** Scatterplot based on the regression model of the dependent variable “Age of onset” and the independent variables “Gender” and “(ATTTC)n”. **b** an additional linear regression model (red line) with confidence interval of 95% (red shadow) plotted over the observed (“actual”) values of the dependent variable (z-scores) and their predicted (“fitted”) values. **c** the residuals of the regression model are plotted against the predicted values of the dependent variable. **d** A bee swarm plot summarizing the distribution of SHAP values for each variable of the regression model is shown. Male gender and shorter “(ATTTC)n” have higher impact value in the age of onset prediction model (pink blots) than female gender and longer “(ATTTC)n” (blue dots). Rank of the selected models of age of onset **e** and disease evolution **f** using the dataset reporting the methylated CpG regions with the five most relevant models (lowest BIC and highest *R*^2^) indicated. **g** and **h** The best model for age of onset (*R*^2^ = 0.998, *n* = 8; *p *value < 0.0007) was found to include the variable 3ʹ(ATTTT)n and the CpG regions “chr1:57361330” in DMR3 and “chr1:57360976” in R4. **i** and **j** The best model of disease evolution (*R*^2^ = 0.999, *n* = 7; *p *value < 0.0008) was obtained with the CpG regions “chr1:57367557” in DMR1, “chr1:57362080” in DMR3, and “chr1:57360845” in R4. **k** and **l** The best prediction model for “Age of onset” considering the “(ATTTC)n”, includes two CpG regions “chr1: 57367004” and “chr1: 57365681”, both located in DMR2 (R^2^ = 0.932, *n* = 8). **m** and **n** A significant model of “disease evolution” associated with the independent variable “(ATTTC)n”, and the combination of two different CpG regions “chr1:57370049” and “chr1:57368270”, both located in DMR1 (*R*^2^ = 0.926, *n* = 7)

We sought to assess the clinical relevance of the exact configuration of the complex repeated elements within the ATTTT/ATTTC *DAB1* genomic region. To this aim, we evaluated the presence of possible clinical relationships between “Age of onset” or “Disease evolution” with a series of genetic variables “WT-(ATTTT)n”, “WT total-repeat-length”, “SCA37-5’(ATTTT)n”, “SCA37-(ATTTC)n”, “SCA37-3’(ATTTT)n”, “SCA37-total-repeat-length”) and “Gender” and “Country of origin”. This information was available from 56 SCA37 patients, 26 from Spain and 30 from Portugal (Suppl. Table 11). We performed this analysis for wild-type and SCA37 alleles. For the “Age of onset”, negative linear correlations with the “(ATTTC)n” (*r* = − 0.572; *p* value = 1.452 × 10^–5^; *n* = 56) and “Country of origin” (r = -0.356; *p *value = 7.265 × 10^–3^; *n* = 56) were found (Fig. 5a; Suppl. Table 12). Moreover, the “3ʹ(ATTTT)n” (r = 0.458; *p* value = 2.839 × 10^–2^; *n* = 22) and “Gender” (r = 0.349; *p* value = 9.141 × 10^–3^; *n* = 56) positively correlated with “Age of onset” (Fig. 5a; Suppl. Table 12). Although no significant correlations were found for “Disease evolution” probably due to the small sample size, a moderate positive correlation with “5’(ATTTT)n” (*r* = 0.371; *p *value = 6.142 × 10^–2^; *n* = 29) was detected.

These results suggest the influence of the (ATTTC)n, geographic origin, gender and the 3’(ATTTT)n on the age of onset in SCA37. Remarkably, this is the first evidence of the phenotypic contribution of the 3’(ATTTT)n number size downstream the (ATTTC)n mutation in SCA37.

### SCA37 regression models for predicting age of onset and disease evolution

A series of predictive linear regression models were generated using all possible combinations of up to five variables from the CpG-identified regions. These models were selected based on the significance of their coefficients (*p* value < 0.05) and ranked based on the coefficient of determination (*R*^2^) and the Bayesian information criterion (BIC), which favours simpler models compared to other popular model selection approaches, such as the Akaike information criterion (AIC).

As expected, and confirming our previous observations (Corral-Juan et al. 2018), the regression analysis identified a model of association considering “Age of onset”, “Gender” and the “(ATTTC)n” repeat size (*R*^2^ = 0.438, *n* = 56; *p *value < 0.039) (Fig. 5b–d, Suppl. Table 13) with an equation model as follows:

$$Age\,\,of\,\,onset = - 0.2692 + \left( {0.7053\,\,x\,\,\,\,Gender} \right) + \left( { - 0.5874\,\,\,\,x\,\,\left( {ATTTC} \right)n} \right),$$

using a value of 0 for females and a value of 1 for males in the “Gender” variable. The analysis of the SHAP values of this model resulted in a contribution of 58.76% of the “(ATTTC)n” and 41.24% of “Gender” to the “Age of onset”. Additionally, a significant association between “Age of onset” and the “Country of origin” was found with a low coefficient of determination (*R*^2^ = 0.127, *n* = 56; *p *value < 0.046), but not in combination with other variables (Suppl. Table 13). In contrast, no significant models were found for “Disease evolution” (Suppl. Table 14).

The same analysis was applied considering the differences in methylation frequencies detected in blood samples in the specific genomic SCA37 region flanking the 5’(ATTTT)n–(ATTTC)n–3’(ATTTT)n repeat tracts as additional independent variables.

Sixty-four out of 86 potentially methylated CpG regions were identified. The variables “Disease evolution” and “Age of onset” were considered when available. A series of linear regression models were constructed with all possible combinations of up to three variables to avoid combinatorial explosion and restrain model complexity. Model selection and evaluation criteria were the same as the previous analysis. Regression analysis identified several models of the dependent variable “Age of onset” (Suppl. Table 15) or “Disease evolution” (Suppl. Table 16). To navigate the sheer amount of models that were generated, we ranked those with the highest number of data points based on lowest BIC and highest *R*^2^ (Fig. 5e, f), and selected the best among the top five models for further dominance analysis. The best model for age of onset (R^2^ = 0.998, *n* = 8; *p *value < 0.0007; Fig. 5g, h) was found to include the variable 3’(ATTTT)n (contributing 31.38% to the model) and the CpG regions “chr1:57361330” in DMR3 (contributing 60.31%) and “chr1:57360976” in R4 (contributing 8.32%). The best model of disease evolution (*R*^2^ = 0.999, *n* = 7; *p *value < 0.0008; Fig. 5 i, j) was obtained with the CpG regions “chr1:57367557” in DMR1 (contributing 25.11% to the model), “chr1:57362080” in DMR3 (contributing 28.74%), and “chr1:57360845” in R4 (contributing 46.15%).

Besides these most significant five predictive models, it is important to highlight the “Age of onset” model identified using the “(ATTTC)n” and the two CpG regions “chr1: 57,367,004” and “chr1: 57,365,681” (*R*^2^ = 0.932, *n* = 8; *p value* < 0.011; Fig. 5k, l), both located in DMR2 and which were also identified contributing together in other 7 significant prediction models(Suppl. Table 15; significant prediction models 23, 31, 35, 39, 43, 45 and 64). Moreover, the analysis also revealed a model of “Disease evolution” associated with the independent variable “(ATTTC)n”, but including the combination of two different CpG regions “chr1:57370049” and “chr1:57368270” both in DMR1 (*R*^2^ = 0.98, *n* = 7; *p *value < 0.0027; Fig. 5 m and 5n). These mathematical predictive models establish the basis for a first attempt towards individualized medicine in SCA37.

### Variable importance identified in linear regression models in SCA37

**Fig. 6:**
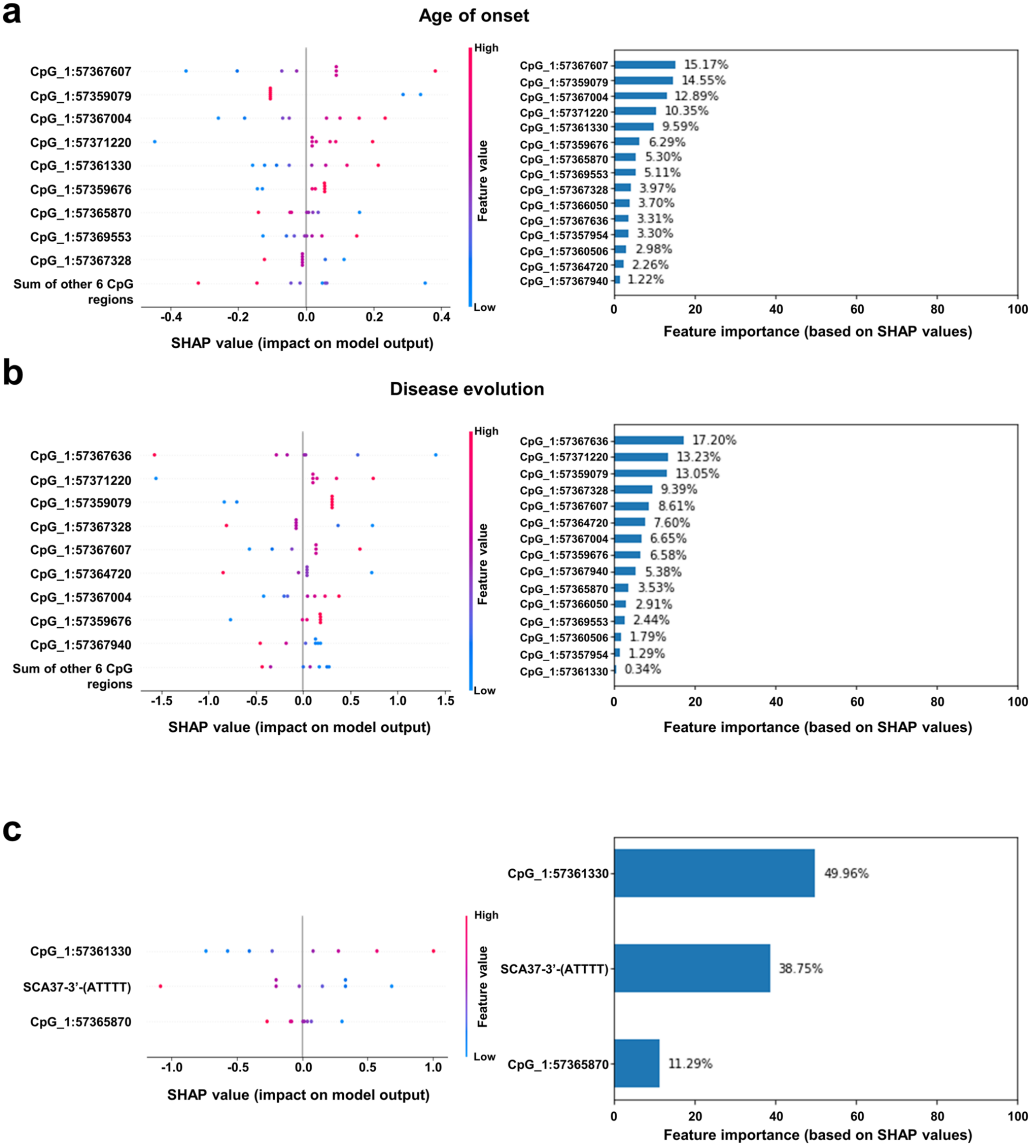
Importance value and impact of methylated CpGs regions in predictive models. SHAP values and relative importance of the 15 most recurrent methylated CpG regions in regression models of “Age of onset” **a** and “Disease evolution” **b**. **c** SHAP values and relative importance of a model of age of onset using 3ʹ(ATTTT)n and CpG regions “chr1:57365870” in DMR2 and “chr1:57361330” in DMR3 (See Fig. 5e)

Considering “Age of onset” and “Disease evolution” associated with methylation signatures, we analysed each variable importance based on SHAP values after selecting the top 15 best predicting CpG regions based on F-regression, which is a rapid linear model for assessing the potential impact of individual regressors one by one, in a sequential manner, without incurring into combinatorial explosion. Prediction models of “Age of onset” and “Disease evolution” using these 15 genomic methylated signatures as independent variables were created and their relative importance based on SHAP values were calculated (Fig. 6a, b). The most important methylation CpG region in the model of “Age of Onset” identified is “chr1:57367607” in DMR1 (15.17%), followed by “chr1:57359079” in R4 (14.55%), while that in the model of “Disease evolution” is “chr1:57367636” (17.20%) and “chr1:57371220” (13.23%) both in DMR1. While the other CpG regions may not seem important in a model solely based on methylation signatures, they became highly prominent when combined with other clinical variables. In particular, CpG regions “chr1:57361330” in DMR3 and “chr1:57365870” in DMR2, significant differentially methylated in SCA37 cerebellar alleles and included in the list of important features for methylation models, also emerged in the top five models for “Age of onset” when utilizing variables of any type (Fig. 5e), such as a model of age of onset involving both CpG regions “chr1:57361330” (49.96%) and “chr1:57365870” (11.29%), and the “3’(ATTTT)n” repeat tract (38.75%) (Fig. 6c; Suppl. Table 15). The analysis of the top 15 best predicting CpG regions (Fig. 6a, b) in the JASPAR transcription factor database revealed four transcription factor binding sites at the same strand and transcription synthesis direction from the *DAB1* gene for the NOBOX, PRDM1, PAX4 and LHX3 transcription factors. Strikingly, PRDM1 transcription factor was found to interact with 82Q ATXN1, the mutant protein in spinocerebellar ataxia type 1 (SCA1), and has been found to be expressed at the granule and Purkinje cells of the cerebellum during chicken embryonic and germline development (Lim et al. 2006; Wan et al. 2014b). This supports the widely accepted hypothesis that common molecular signalling alterations underlie cerebellar neurodegeneration in spinocerebellar ataxias.

### Common origin and age of the SCA37 (ATTTC)_n_ mutation

**Fig. 7:**
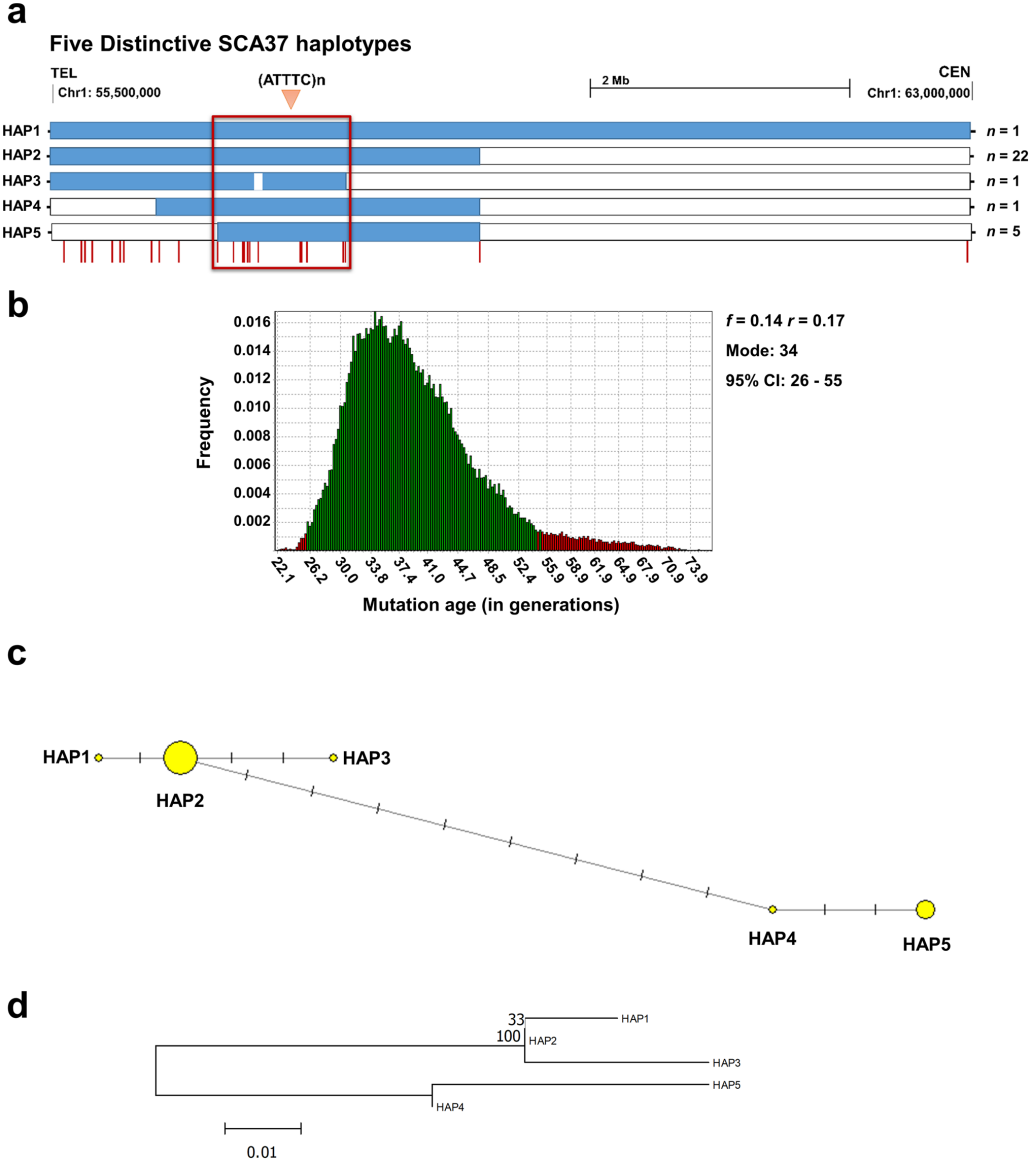
Distinctive SCA37 haplotypes and common origin of the SCA37 mutation. **a** Haplotype analysis revealed the presence of a 964 kb shared region (red box) in all Iberian SCA37 patients segregating with the causative SCA37 mutation, revealing a common origin of the SCA37 mutation in the Iberian Peninsula which originated approximately 859 years ago (95% CI: 647–1378). Red bars represent informative SNPs positions. **b** DMLE + 2.3. analysis showing a posterior probability density of the mutation age for population grown rate (*r* = 0.17) and the proportion of sampled disease-bearing chromosomes (*f* = 0.14) considering an intergenerational time interval of 25 years. The estimated median age identified is 34 generations. Green bars show the 95% confidence interval between 26 and 55 generations. The frequency at which each number of generations was resulted from the iterations is shown on the y-axis. Outcome considering the total or the southwest of the Iberian Peninsula population with either 20 or 25 years/generation are included in Fig. S10 of the Additional file 2. **c** Haplotype network showing the phylogenetic relationship among five different SCA37 pathogenic alleles. Circle size is proportional to the number of chromosomes; line length is proportional to the genetic distance among haplotypes. **d** Phylogenetic reconstruction based on genetic distances (D_A_) between the five haplotypes. The numbers next to nodes, represent a measure of support for the node. The line bar with 0.01 value indicates the number of genetic changes (nucleotide substitutions per site)

Long-read sequencing of targeted enriched CRISPR–Cas9 SCA37 expanded alleles including the (ATTTC)n repeat mutation and the flanking regions from 11 Spanish patients from eight unrelated kindreds confirmed a common haplotype spanning a 22.29 kb region within intron 11 of the *DAB1* gene (Suppl. Table 17). A combination of SNP-genotyping and PHASE inference of 24 SNPs adjacent to the (ATTTC)n expanded insertion was used to elucidate a possible SCA37 ancestral haplotype. Additional data from four SCA37 Spanish and 16 Portuguese patients from 14 unrelated kindreds were also included for haplotype inference. Haplotype reconstruction revealed a 964-kb shared region in linkage disequilibrium flanking the (ATTTC)n mutation in SCA37 chromosomes in all SCA37 patients (Fig. 7a; linkage disequilibrium *p *value < 0.00001; Suppl. Table 17), evidencing a common origin of the SCA37 mutation in the Iberian Peninsula. The patient I:1 AT-F SP-F1 individual shared the complete haplotype except for SNP rs954450605, indicating that a single-nucleotide substitution occurred later in this marker. A total of five distinctive haplotypes were identified in all 30 SCA37 Iberic patients (Fig. 7a). Twenty-two patients from five Spanish and four Portuguese kindreds shared the most frequent haplotype in the cohort spanning 3.14 Mb in the *DAB1* genomic region (Fig. 7a). Considering a disease prevalence of *f* = 0.14, an Iberic population growth rate of *r* = 0.17, and considering each generation to span 25 years, the mutation was estimated to have occurred 859 years ago (95% CI 647–1,378; Fig. 7b). The estimation of the mutation age considering different demographic and disease prevalence parameters for the DMLE + 2.3 software generated comparable results (Suppl. Figure 11). Likewise, considering the southwest of the Iberian Peninsula instead of the whole Iberic region, the mutation was estimated to have occurred approximately 917 years ago (95% CI: 656–1404) (Suppl. Figure 11). Phylogenetic relationship among five different SCA37 haplotypes were generated showing the most parsimonious relationship between them considering 24 informative SNPs in 14 SCA37 families (Fig. 7c, d). A common haplotype (HAP2) was shared by five Spanish and four Portuguese families. In the Spanish AT-901 family this haplotype resulted from a recombination event from HAP1 haplotype (Supplementary Table 17; (Corral-Juan et al. 2018)) HAP3 and HAP4 haplotypes correspond to one Spanish and one Portuguese families, respectively. Relevantly, HAP5 haplotype is shared by two Spanish and one Portuguese families and appeared from a recombination event in the Spanish AT-9012 family (Supplementary Table 17; (Corral-Juan et al. 2018)).

## Discussion

In the present study, we accurately determine the pathogenic *DAB1* 5ʹ(ATTTT)n–(ATTTC)n–3’(ATTTT)n repeat tract size and configuration in SCA37 using an unbiased non-PCR amplified long-read sequencing of the CRISPR–Cas9 targeted enriched locus. By sequencing long fragments of native gDNA spanning the *DAB1* repeat expansion, we were able to identify a differential SCA37 methylation signature in SCA37 alleles in cerebellum. We could also establish novel genotype–phenotype associations considering molecular and disease’s variables such as “Age of onset” and “Disease evolution” by generating predictive regression models in SCA37. Long-read sequencing of the (ATTTC)n genomic region in combination with 24 SNPs genotyping proved a common haplotype spanning a 964 kb genomic region within *DAB1* intron 11 in all SCA37 expanded alleles from 30 Spanish and Portuguese SCA37 patients demonstrating a common origin of the SCA37 mutation in the Iberian Peninsula. The SCA37 mutation was estimated to have occurred 859 years ago (95% CI 647–1,378).

To date, long-PCR followed by Sanger sequencing for alleles with repeats of moderate size has been the method of choice for genetic diagnosis of SCA37 (Matilla-Dueñas and Volpini 1993). However, this is challenging when sequencing long repeat tracts. By using unbiased non-PCR amplified nanopore sequencing and CRISPR–Cas9 targeted enrichment of the SCA37 mutation region we could unequivocally size and determine the configuration of the 5ʹ(ATTTT)n, (ATTTC)n and 3ʹ(ATTTT)n repeat tracts, identifying a novel association between disease age of onset and the 3´(ATTTT) repeat size in SCA37 alleles. This highlights the importance of accurately determining the exact configuration of the pathogenic alleles to establish accurate genotype–phenotype correlations in SCA37. Importantly, we detected the highest sequencing coverage when fresh high molecular weight DNA was obtained during gDNA extraction. This is of relevance for obtaining consistent accurate repeat sequences and methylation signatures by nanopore sequencing.

Tandem repeat expansions (TREs) have been described to be causative of at least 60 human monogenic disorders, including psychiatric, neurodevelopmental, neuromuscular, and neurodegenerative disorders (Wen et al. 2023). Implementing an accurate method for sequencing long-tandem repeats without PCR bias removes experimental variability, overcoming the technical limitations for genetic diagnosis of those disorders and enabling genotype–phenotype correlations needed in precision medicine. A recent study has described the first SCA37 patients identified outside the Iberian Peninsula by using biased long-range PCR followed by long-read nanopore sequencing to determine the repeat size and structure of the 5ʹ(ATTTT)n–(ATTTC)n–3ʹ(ATTTT)n repeat tract. In this case, the exact size of the repeat could not be precisely determined due to PCR amplification bias and high experimental variability (Rosenbohm et al. 2022). In contrast, in the present study targeted CRISPR–Cas9 enrichment of the native DNA including the SCA37 repeat region in combination with long-read nanopore sequencing obtained an average base accuracy rate of 96.4% with a mean sequencing error of 3.6% (range: 3.01–4.62%) for an average 521-fold enrichment. By avoiding bias from PCR amplification, our strategy notably reduced repeat count variability compared to other studies (Rosenbohm et al. 2022). Another important limitation of nanopore sequencing of PCR amplicons containing TREs is that it cannot reveal allele-specific methylation signatures such as the ones identified in our study.

Remarkably, we found that the 5ʹ(ATTTT)n–(ATTTC)n–3ʹ(ATTTT)n repeat tracts in all SCA37 alleles were preceded by an ATTTTTTT sequence. In contrast, the (ATTTT)n repeat tract in wild-type alleles were found to be preceded by ATTTATTT. ATTT motifs are switch regulatory elements (SREs), and were identified as tetrameric targets of POU-family transcription factors located on promoter regions acting as cis-regulatory elements repressing gene transcription (Schaffer et al. 2003; Alazard 2005; Lachman et al. 2006). Moreover, TTTATTTA sequences form highly compact native DNA structures called mini-dumbbells (MDBs) implicated in stabilizing DNA structure potentially affecting protein binding and DNA translation and replication (Guo and Lam 2016). Likewise, replacement of the TTTATTTA sequence by ATTTTTTT in SCA37 alleles would influence DNA secondary structure providing repeat instability and transcription dysregulation.

Repeat interruptions contribute to stabilizing repeat tracts of non-pathogenic alleles during somatic and germinal transmission (i.e. SCA1, SCA2, HTT, etc.)*,* whereas in *RFC1*, *SCA10*, *SCA27B* and *SCA31* genes, interruptions are present in expanded alleles where the effects on repeat stability are unclear (Moseley 2000; Richards 2001; Sobczak and Krzyzosiak 2005; Wright et al. 2019; Wilke et al. 2023). In our study, all the (ATTTT)n and (ATTTC)n repetitive motifs in SCA37 chromosomes were uninterrupted, whereas one out of 29 of wild-type alleles presented AT-rich interruptions such as (ATTTT)_2_AT(ATTTT)_20_A(ATTTT)_22_(ATTT)(ATTTT)_4_(ATTT)(ATTTT)_2_ similar to the one previously reported (Loureiro et al. 2019).

In most of the repeat expansion diseases, the expansion is unstable during somatic and germline transmissions triggered by DNA synthesis or repair errors (Matsuura et al. 2004; Kacher et al. 2021). Those errors apply for most of instable repeat tracts, depending on repeat size, configuration, genomic location or cell type (Khristich and Mirkin 2020; Mouro Pinto et al. 2020). In the present study, the analysis of the different repeat motifs conforming the 5ʹ(ATTTT)n–(ATTTC)n–3ʹ(ATTTT)n in SCA37 alleles revealed differential repeat instability when compared in blood, cerebellar, and fibroblasts cells. Cerebellar samples from two SCA37 patients presented higher level of somatic instability of the 5’(ATTTT)n–(ATTTC)n–3ʹ(ATTTT)n repeat tract, with an increase in the number of (ATTTC)n, and a decrease of the 5ʹ(ATTTT)n and 3ʹ(ATTTT)n repeats, compared to PBLs. No instability was observed when comparing WT-(ATTTT)n alleles in the three different cellular types. Relevantly, fibroblasts cells presented a decreased number of all three pentanucleotide motifs in the SCA37 repeat tract in pathogenic alleles when comparing with PBLs. Tissue-specific somatic instability has been identified in a few TREs associated with other neurodegenerative diseases such as Huntington’s disease, where the CAG repeat shows a lower instability in spinal cord and cerebellum, or SCA1 and SCA3 with a lower degree of mosaicism found in the cerebellar cortex for the CAG repetitive tracts compared to other CNS regions (Cancel et al. 1998; Kraus-Perrotta and Lagalwar 2016; Mouro Pinto et al. 2020). In contrast, *FXN* GAA expanded repeats in Friedreich’s ataxia patients show an expansion bias in the cerebellum (De Biase et al. 2007), and skeletal muscle in Myotonic Dystrophy Type 1 present with much larger *DMPK* CTG expansions (Thornton et al. 1994). In the present study, somatic instability revealed tissue-specificity instability of the 5ʹ(ATTTT)n–(ATTTC)n–3ʹ(ATTTT)n repeat tract in the affected SCA37 cerebellum. It appears that somatic instability depends on the type and length of the expanded repeat, increases with age, and is also influenced by DNA replication and the repair genes implicated in each particular cell type (Pearson et al. 2005).

A relevant and novel contribution of the present study is the identification of specific methylation signatures in SCA37 disease alleles that are supportive of dysregulated expression of the *DAB1* gene observed in SCA37 cerebellum (Corral-Juan et al. 2018). Likewise, Fragile X-associated tremor/ataxia syndrome (FXTAS) is characterized by cerebellar ataxia and presents with an unmethylated repeat expanded mutation leading to increased expression of the gene product and Purkinje cell loss in the cerebellum (Kenneson 2001).

We generate for the first time a predictive mathematical model for the age of onset and diseases evolution in SCA37 considering the importance of genetic variables such as the size and configuration of the complex 5ʹ(ATTTT)n–(ATTTC)n–3ʹ(ATTTT)n repeat tract. To the best of our knowledge there are only a few studies using mathematical models and machine learning algorithms to predict clinical outcomes in other spinocerebellar ataxias including SCA1, SCA2, SCA3 and SCA6 (Tezenas du Montcel et al. 2014; Peng et al. 2021; Ru et al. 2022; Hatano et al. 2023). Predictive outcome models are becoming very useful tools for genetic counselling, clinical prognosis, and response follow-up of therapeutic treatments.

In this study, we have identified an identical 964 kb haplotype found in linkage disequilibrium flanking the (ATTTC)_n_ repeat inserted mutation (chr1:56785142–57749488, hg38) shared by all SCA37 patients studied from 14 Spanish and Portuguese kindreds, indicating that an ancestral chromosome is responsible for all SCA37 cases in the Iberian Peninsula described to date. Common ancestral origins and mutation founder effects have been described in several SCAs (Verbeek et al. 2004; Sequeiros et al. 2012), such as SCA2 in Cuba (Velázquez Pérez et al. 2009), SCA3 in Azores (Gaspar et al. 2001) or SCA36 in the Spanish Costa da Morte (García-Murias et al. 2012). The relatively high frequency of the SCA37 mutation in the south of the Iberian Peninsula, compared to other SCAs, has implications for disease prioritization during genetic diagnosis. We were able to trace back the relatively recent origin of the (ATTTC)_n_ pathogenic insertion to 1164 (645–1376, 95% CI). Estimating the age of mutations partly depend on parameters that are difficult to exactly determine such as population growth rate or proportion of disease-bearing chromosomes (Rannala and Bertorelle 2001). Although these are possible limitations of this study, there is consistency in the resulted estimated age when using two different intergenerational time intervals giving reliability to our data (Fenner 2005). It would be interesting to investigate whether the SCA37 mutation found in German SCA37 patients (Rosenbohm et al. 2022) and in the Iberian Peninsula SCA37 patients share the same common haplotype. Such is the case for the SCA3/Machado-Joseph disease CAG pathogenic repeat, where a common origin chromosome was found in most families worldwide (Bettencourt et al. 2008). Interestingly, by the time the SCA37 mutation originated according to our study, the southwest region of the Iberian Peninsula comprised a part of al-Andalus occupied by a significant Arabic and North African populations. Therefore, we cannot rule out a non-European origin of the original SCA37 chromosome.

## Conclusions

In this study, by accurately determining the size and configuration of the complex 5ʹ(ATTTT)n–(ATTTC)n–3ʹ(ATTTT)n repeat tract within the *DAB1* gene underlying SCA37 pathology by CRISPR/Cas9-mediated enrichment combined with nanopore long-read sequencing, we establish novel genotype–phenotype associations with significant implications for genetic diagnosis. Importantly, we also identify differential cerebellar hypomethylation upstream the repeat in SCA37 alleles that may account for DAB1 pathogenic cerebellar dysregulation in SCA37. Finally, this study provides evidence of a relatively recent common origin of the SCA37 mutation in the Iberian Peninsula dated to 1164 CE.

### Supplementary Information

Below is the link to the electronic supplementary material.

Supplementary file1 (PDF 2056 KB)

Supplementary file2 (XLSX 8559 KB)

Supplementary file3 (PDF 299 KB)

## Data Availability

The data generated and analysed during the current study are available from the corresponding author on reasonable request.

## Abbreviations

AIC: Akaike information criterion
BIC: Bayesian information criterion
CB: Cerebellum
DAB1: Disabled-1
DMF: Differentially methylated frequency
DMR: Differentially methylated region
FC: Fibroblasts cells
GPU: Graphics processing unit
HAC: High accuracy
HMW: High molecular weight
IGV: Integrative genomics viewer
IQR: Interquartile range
LLR: Log-likelihood ratio
NGS: Next-generation sequencing
OLS: Ordinary least squares regression
PBL: Peripheral blood leukocytes
SCA: Spinocerebellar ataxia
SCA37: Spinocerebellar ataxia type 37
SD: Standard deviation
SHAP: SHapley additive exPlanations
SNP: Single-nucleotide polymorphism
SUP: Super accuracy
TRE: Tandem repeat expansion
