# Supplementary material for: Novel genotype–phenotype correlations, differential cerebellar allele-specific methylation, and a common origin of the (ATTTC)n insertion in spinocerebellar ataxia type 37
Source: Hum Genet. 2024 Feb 23;143(3):211–32. doi: 10.1007/s00439-024-02644-7 (PMC11043136; doi:10.1007/s00439-024-02644-7)
Supplement: Supplementary file 1 — Supplementary file1 (PDF 2056 KB) [file 439_2024_2644_MOESM1_ESM.pdf]

Novel genotype-phenotype correlations, differential cerebellar allele-specific methylation, and a common origin of the $(ATTTC)_n$ insertion in spinocerebellar ataxia type 37

Human Genetics

Marina Sanchez-Flores[1†], Marc Corral-Juan[1†], Esther Gasch-Navalón[1], Davide Cirillo[2], Ivelisse Sanchez[1], Antoni Matilla-Dueñas[1*].

1. Functional and Translational Neurogenetics Unit, Department of Neuroscience, Research Institute Germans Trias i Pujol (IGTP), Universitat Autònoma de Barcelona-Can Ruti Campus, Badalona, Barcelona, Spain.
2. Barcelona Supercomputing Center (BSC), Barcelona, Spain.

†Marina Sanchez-Flores and Marc Corral-Juan have contributed equally to this work.

*To whom correspondence should be addressed at:

Dr. Antoni Matilla-Dueñas

Head of the Neurogenetics Unit

Health Sciences Research Institute Germans Trias i Pujol (IGTP)

Carretera de Can Ruti, Camí de les Escoles s/n

08916 Badalona, Barcelona, Spain

Telephone: +34 930 330 532

Fax: +34 934 978 654

E-mail: amatilla@igtp.cat

PCR amplification and sequencing of normal and SAC37 repeat alleles in the *DAB1* gene

The pentanucleotide repeats were amplified by standard PCR with primers listed in Table S1 using the LA Taq DNA polymerase (TaKaRa, Cat. no. RR002M). Every reaction contained 150 ng of gDNA, 0.16 µM of each primer in a reaction volume of 25 µL. After 4 min at 94 ºC, DNA samples underwent 20 cycles of amplification (94 ºC for 30 sec, 58 ºC for 2 min, 72 ºC for 2 min, each) followed by additional 30 cycles (94 ºC for 30 sec, 58 ºC for 2 min with 15 sec increment per cycle, 72 ºC for 2 min, each). PCR products were separated by electrophoresis in 1% agarose gel, both alleles were excised from the gel and purified using NucleoSpin Gel and PCR Clean-up kit (MACHEREY-NAGEL GmbH & Co. KG, Cat. no. 740609.50) following the manufacturer's protocol eluting in 20 µL. Purified products were directly Sanger sequenced using the BigDye® Terminator v3.1. (Thermo Fisher Scientific, Cat. no. 4347455) with 24F and 24R internal primers (Table 1). Sequencing PCR conditions were as follows: an initial heat denaturation step of 95 °C for 5 min, followed by 50 cycles of 95 °C for 30 sec, 55 °C for 10 sec, and 60°C for 4 min, each. Capillary electrophoresis was performed with an ABI Prism 3130 Genetic Analyser (Thermo Fisher Scientific, Cat. no. 3130XLR) and sequences were analysed with Sequence Scanner v1.0 (Thermo Fisher Scientific).

Multiple-guide CRISPR/Cas9 targeted enrichment

Up to seven µg of gDNA were dephosphorylated using 15 units of quick calf intestinal phosphatase (NEB, Cat. no. M0525S) in 30 µL of 1xCutSmart buffer (NEB, Cat. no. B7204S) for 10 min at 37 ºC to prevent adapter ligation with the non-target DNA fragment ends, followed by heat inactivation for 2 min at 80 ºC. Six crRNA sequences (IDT, Custom designed) were designed on complementary strands flanking the (ATTTC)n SCA37 mutation spanning a total target size of 22.29 kb. Sequences are provided in Table 2. Sequences were designed with the CHOPCHOP tool (www.chopchop.cbu.uib.no) on opposite strands to have paired cleavage sites at the 5' and 3' flanking SCA37 mutation. Stringent prefiltering conditions were applied to achieve the best Cas9 cleavage efficiency: GC content between 35 and 60% (1), crRNA with self-complementarity (SC) score of zero (2), efficiency scores >0.4, and number of mismatches (MM) between the crRNA and the target DNA sequence: MM0 ≤ 1, MM1 ≤ 1, MM2 ≤ 1 and MM3 ≤ 5. Candidate crRNA sequences from the CHOPCHOP tool were checked with the IDT online tool

(https://eu.idtdna.com/site/order/designtool/index/CRISPR_SEQUENCE) to select crRNA sequences with the maximum on-target and minimum off-target activity. A 100 µM mixture of the six Alt-R® CRISPR-Cas9 crRNAs (10 µM each) and 100 µM of Alt-R® CRISPR-Cas9 trans-activating (tracrRNA) (IDT, Cat. no. 1073189) were combined to prepare crRNA:tracrRNA duplexes by 5 min at 95 ºC and cooling down on benchtop for 5 min. Subsequently, 10 pmol of the crRNA:tracrRNA duplexes were combined with 10 pmol Alt-R® S.p. HiFi Cas9 Nuclease V3, 100 µg (IDT, Cat. no. 1081060) in 1xCutSmart® Buffer (NEB, Cat. no. B7204S) to a final volume of 30 µL and incubated at RT for 30 min to form ribonucleoprotein (RNP) complexes. Dephosphorylated gDNA was cleaved by adding 12 µL of the RNP complexes and incubation at 37 ºC for one hour and, then, Cas9 was heat inactivated 5 min at 72 ºC for its release from DNA cleaved ends. Moreover, 120 units of thermolabile proteinase K (PK) (NEB, Cat. no. P8111S) were added to ensure that no Cas9 remained attached to DNA, which might interfere with adapters ligation, by 15 min of incubation at 37 ºC, PK was then inactivated at 55 ºC for 10 min as recently suggested by Keraite and collaborators (3).

Cas9 editing efficiency determination by quantitative real time PCR (qRT-PCR)

Editing efficiencies of the CRISPR-Cas9 RNP complexes by qRT-PCR in each Cas9 cleavage site. Three controls, two located 100 bp outside of the targeted region and one additional control within exon 1 of the *GAPDH* gene, were used to represent background DNA to normalize amplified DNA levels at the cleavage site by the $2^{-\Delta\Delta C}$T method as specified by Li *et al.*, 2019 (4). Normalized ratios of cleaved and non-cleaved samples were divided and subtracted to 1.

qRT-PCR assays were carried out with 1 U/µL of the TB Green® Premix Ex Taq™ (Tli RNaseH Plus) (TaKaRa Bio, Cat. no. SD3033) and 0.4 µM of each primer in a final volume of 10 µL. In a 96-well plate, three technical replicates of each sample were amplified using the LightCycler® 480 (Roche Diagnostics). Reactions were subjected to an initial denaturation step of 95 ºC for 2 min, and 40 cycles of 95 ºC for 5 sec, 60 ºC for 20 sec, 72 ºC for 20 sec each.

Library preparation for long-read nanopore sequencing

Enriched SCA37 samples were dA-tailed by adding 1 µL of 10 mM dATPs (Zymo research, Irvine, CA, Cat. no. D1005) and 1 µL of Taq polymerase (NEB, Cat no. M0267) for 5 min at 72 ºC. A-tailed samples were ligated with the AMX sequencing adapters

using the T4 DNA Ligase (NEB, Cat no. E6057) for 10 min at RT. Unligated adapters and short DNA fragments were then removed by 0.3xAMPure XP beads (Beckman Coulter, Cat. no. A63880) purification, washing twice with long fragment buffer before eluting 10 min at 37 ºC with 30 µL of elution bBuffer. Finally, 37.5 µL of sequencing buffer and 25.5 µL of loading beads were added to the previously generated DNA library, which was sequenced during 72 hours in a PromethION sequencer

SNP genotyping

PCR reactions were performed using 0.3 µM of forward and reverse primers listed in Table S1 with one unit of recombinant Taq DNA Polymerase (Invitrogen, Cat. no. 100021276) in a total volume of 20 µL. The reactions were subjected to an initial heat denaturation step of 9 5°C for 5 min, followed by 37 cycles of 95 °C for 40 sec, 58 °C for 40 sec, and 72°C for 1 min, each. The final extension step was 72 °C for 10 min. Purified products with the Illustra™ ExoProStar™ 1-STEP Kit (GE Healthcare, Cat. no. US77705) were directly sequenced by Sanger's method using the BigDye® Terminator v3.1. (Thermo Fisher Scientific, Cat. no. 4347455). PCR sequencing reactions were subjected to an initial heat denaturation step of 95 °C for 5 min, followed by 30 cycles of 95 °C for 10 sec, 55 °C for 5 sec, and 60 °C for 4 min, each. Sequencing reactions were run on an ABI Prism 3130 Genetic Analyser (Thermo Fisher Scientific, Cat. no. 3130XLR) and sequences analyzed by Sequence Scanner v1.0 (Thermo Fisher Scientific).

**Primer ID**	**Sequence (5'-3')**
***DAB1* Real Time qRT-PCR**	
Cas9_CS1_FW	AGCCTGAATCCAGAGTCCAA
Cas9_CS1_RV	GGAGATCCAACACTCCCTCA
Cas9_CS2_FW	GGATCTCTTAACATTTGTTCCTCCT
Cas9_CS2_RV	CAGCAATCTCACCACTGGAA
Cas9_CS3_FW	TGAGTTGTATGTATTCACATAGTTCCA
Cas9_CS3_RV	GAACCATAACATTCTCCTTCTCA
Cas9_CS4_FW	TGTTCCTACAAACTGCATCGTT
Cas9_CS4_RV	TGATCTGTAGCATGCAGGTAAAA
Cas9_CS5_FW	ATGGAATCTCTTGCAGCACA
Cas9_CS5_RV	AGCCTAGGTAACGCTGACTG
Cas9_CS6_FW	GGTGATTCATTTTTATGATTAACTGC
Cas9_CS6_RV	TGAAATAAATATCATTTGTGCTTGC
Cas9_CS5_External_ROI_FW	AGATGGCAGTTTTTCAGTAGGC
Cas9_CS5_External_ROI_RV	TTTCCAGCTACCTGTGAACATC
Cas9_CS6_External_ROI_FW	GGGCTGCTGGTCTATTTGTT

Cas9_CS6_External_ROI_RV	GAAACTGAAGGAGTAAGATTTGAACC
**GAPDH Real Time qRT-PCR**	
GAPDH_FW	ACGTAGCTCAGGCCTCAAGACCT
GAPDH_RV	TTCTCTCCGCCCGTCTTCAC
**WT-(ATTTT)n / (ATTTC)n repeat PCR amplification**	
(ATTTT)n_FW	GAATCCATGCTACTGGCTGCA
(ATTTT)n_RV	AGGCACCTAGCTCTGATTAGAAACTTT
**WT-(ATTTT)n / (ATTTC)n repeat sequencing**	
24F	GAAGTGGTCCTCCCAAGTCA
24R	GAAAGAATGAAGCCCAGGAA
**Genetic polymorphic markers on 1p32-1p31 for common origin determination**	
rs149218089_FW	CAAAACCTCTTCCTCTGAGACAA
rs149218089_RV	TGAAAACCATGGTACCAAAAGA
rs954450605_FW	GCCCAGCATTTTATTGCCTA
rs954450605_RV	GGGGTGGCAGAGTTCAGTAA
rs929412570_FW	GAATGGGGATCATCACTCAAA
rs929412570_RV	GCAGTTAGCTTGGCACTTGA
rs76443397_FW	AGAAATGTAACTCAAACTAGGACCA
rs76443397_RV	TGCAAGCAACATAAATGGACTC
USP1_EX9_FW	GCTAATTTTGAACCTGAAACTTTGTG
USP1_EX9_RV	TCCTACTATTACAGCATTTTGAACAAGAC

Suppl. Table 1 List of primer sequences used.

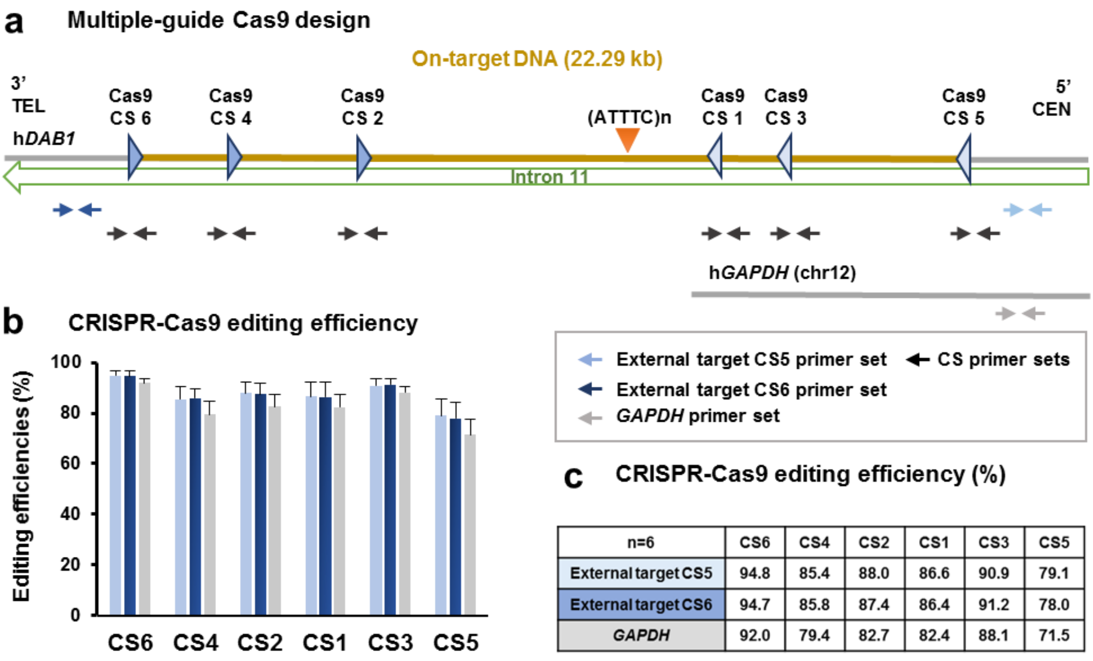

Suppl. Figure 1 a Schematic representation of the six Cas9 cleavage sites (CS) and designed primer sets for intron 11 of the *DAB1* gene (green arrow) and *GAPDH gene*. Cleavage sites in light blue if located upstream the (ATTTC)$_n$ inserted repeat (orange

triangle) or dark blue if located downstream (5’ to 3’ gene sense). The 22.29 kb on-target DNA corresponding to the farthest cleavage sites is indicated in dark yellow. TEL, telomere; CEN, centromere. **b** Multiple-guide CRISPR/Cas9 editing efficiencies obtained by qRT-PCR amplification of the six DAB1 cleavage sites in four SCA37 and two control samples (n). Bars denote the standard error of the mean (SEM). **c** Summary table of the editing efficiency percentages. Efficiencies were calculated by normalizing amplification values with the *GAPDH* (grey), the CS5 external target (light blue) or the CS6 external target (dark blue) controls. Editing efficiencies were calculated as the ratio of the cleaved and control sites of the CRISPR/Cas9 treated and non-treated samples represented as a percentage with the standard error of the mean (SEM).

**crRNA ID**	**Sequence (5'-3')**
**CRISPR/Cas9 target enrichment**	
crRNA_Cas9_CS_1	TGTATATCTTGATACTACGT
crRNA_Cas9_CS_2	TATATCCCCTCACTAACATG
crRNA_Cas9_CS_3	GGATGTGGGATACTCGACGG
crRNA_Cas9_CS_4	GGTCATCCAGCACATATCAG
crRNA_Cas9_CS_5	ATATCAAACTTCCTTACGAC
crRNA_Cas9_CS_6	ATACTGAGTTATTCACGCTT

Suppl. Table 2 List of CRISPR-RNAs (crRNAs) sequences used.

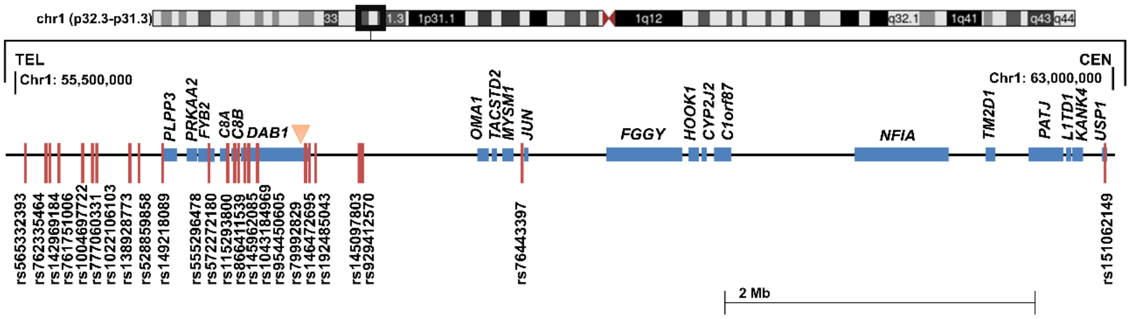

Suppl. Figure 2 Genomic representation of the SNPs considered for common origin determination within the 6.8 Mb 1p32-1p31 region spanning the (ATTTC)n expanded insertion in the *DAB1* gene. Vertical red bars represent the 24 selected genetic markers; blue boxes represent coding exons of the genes present in the region; orange triangle represents the (ATTTC)n mutation within the *DAB1* gene.

Ped.	Ped. ID	Gender	Age of onset	Disease evolution	Sample	Type of sample	Age at collection
AT-901	IV:9	Female	55	27	SPA001	Blood	75
						Cerebellum	78
	IV:10	Female	39	38	SPA002	Blood	65
						Cerebellum	67
	IV:4	Male	64	15	SPA003	Fibroblasts	74
AT-9012	III:3	Female	43	27	SPB001	Blood	62
	IV:9	Male	NA	NA	SPB002	Blood	NA
AT-59	IV:9	Female	32	19	SPC001	Blood	48
						Fibroblasts	50
AT-90	III:6	Male	46	4	SPD001	Blood	50
AT-E	I:1	Male	40	NA	SPE001	Blood	NA
AT-F	I:1	Female	42	11	SPF001	Blood	53
AT-G	I:1	Female	37	10	SPG001	Blood	47
AT-H	I:1	Female	NA	NA	SPH001	Blood	NA

Suppl. Table 3 Clinical features of the 11 affected SCA37 nanopore sequenced individuals from eight independent Spanish kindreds and their age at collection of blood, fibroblast and/or cerebellar samples.

crRNA ID	Chromosomal localization	Strand	Target sequence	PAM	GC (%)	CHOPCHOP tool SC	MM 0	MM 1	MM 2	MM 3	Efficiency	IDT On-target	Off-target
crRNA_Cas9_CS_6	Chr1: 57,353,671-57,353,690	+	ATACTGAGTTATTCACGCTT	AGG	35	0	1	0	0	2	42.12	63	68
crRNA_Cas9_CS_4	Chr1: 57,356,511-57,356,530	+	GGTCATCCAGCACATATCAG	GGG	50	0	1	0	0	5	71.39	35	59
crRNA_Cas9_CS_2	Chr1: 57,359,850-57,359,869	+	TATATCCCCTCACTAACATG	TGG	40	0	1	0	0	4	63.26	66	59
crRNA_Cas9_CS_1	Chr1: 57,369,331-57,369,350	-	TGTATATCTTGATACTACGT	GGG	30	0	1	0	1	3	69.53	72	75
crRNA_Cas9_CS_3	Chr1: 57,371,254-57,371,273	-	GGATGTGGGATACTCGACGG	TGG	60	0	1	0	0	0	74.59	52	90
crRNA_Cas9_CS_5	Chr1: 57,375,944-57,375,963	-	ATATCAAACTTCCTTACGAC	AGG	35	0	1	0	0	3	51.35	68	83

Suppl. Table 4 crRNA sequences designed for CRISPR/Cas9-mediated sequence cleavage. (SC) Self-complementarity; (MM) number of mismatches.

Sample ID	Sample type	DNA extraction	Whole genome						CRISPR/Cas9-target region				SCA37-5'(ATTTT)n-(ATTTC)n-3'-(ATTTT)n region		
			Number of reads	Mean read length	Read length N50	Mean read quality	Number of primary aligned reads	Mean genome-wide depth of coverage	Error rate (%)	Number of reads	Mean depth of coverage	Enrichment increment	Number of reads	Mean depth of coverage	Enrichment increment
**SPA001**	PBL	Standard	289,005	4,692	11,513	12.8	230,675	0.38	3.09	1281	206.08	545.95	245	199.03	527.24
**SPA001**	CB	HMW	255,986	8,710	28,859	12.7	208,951	0.64	3.58	1567	354.13	551.61	504	401.63	625.72
**SPA002**	PBL	Standard	192,977	2,700	7,871	10.0	125,778	0.10	3.87	196	29.21	292.20	38	32.93	329.57
**SPA002**	CB	HMW	197,261	9,995	24,322	12.9	160,837	0.57	3.71	1617	305.72	531.66	477	382.17	664.87
**SPA003**	SF	HMW	151,559	12,045	39,599	12.9	127,214	0.51	3.35	2168	464.97	914.11	684	531.01	1044.10
**SPB001**	PBL	Standard	49,293	4,652	16,362	8.5	22,540	0.05	4.62	22	8.43	158.42	12	8.76	164.63
**SPB002**	PBL	HMW	372,104	5,820	21,213	12.7	300,334	0.59	3.24	1011	189.08	319.68	245	211.47	357.64
**SPC001**	PBL	Standard	61,997	3,663	9,407	8.9	35,430	0.05	3.01	336	46.53	915.77	48	47.85	941.78
**SPC001**	SF	HMW	231,169	10,219	33,529	13.3	197,835	0.70	3.47	3521	708.25	1,015.18	948	946.80	1357.45
**SPD001**	PBL	Standard	34,617	3,500	10,627	8.4	18,924	0.03	3.21	315	47.17	1,744.71	57	54.12	2002.00
**SPE001**	PBL	Standard	2,184,017	1,911	2,719	13.7	1,974,997	1.15	3.59	462	46.34	39.45	36	32.60	27.45
**SPF001**	PBL	Standard	2,761,739	2,961	4,587	13.6	2,531,627	2.39	4.05	397	58.89	23.60	70	69.51	28.04
**SPG001**	PBL	Standard	34,840,588	1,559	2,205	13.7	32,353,133	15.77	3.37	495	38.60	1.45	27	25.92	0.64
**SPH001**	PBL	Standard	809,902	1,574	2,221	13.7	747,388	0.35	4.18	499	85.60	241.74	88	78.92	222.80
		**Mean**	**3,030,872**	**5,286**	**15,360**	**12.0**	**2,788,262**	**1.66**	**3.60**	**991.929**	**184.93**	**521.11**	**248.5**	**215.91**	**592.42**

Suppl. Table 5 Summary of the Cas9 enrichment and the long-read sequencing performance for the 14 nanopore sequenced DNA sample. CB, cerebellum; FC, Fibroblasts cells; HMW, high molecular weight; PBL, peripheral blood. Number of reads includes WT, SCA37 and non-classified reads.

Chr	Starting coordinate	Ending coordinate	Enriched samples	Mean depth in enriched samples	Type of region	Position
chr1	13,470,000	13,475,000	7	26.88	crRNA_Cas9_CS4 off-target (6MM)	chr1:13,471,615
chr1	57,365,000	57,370,000	13	255.44	Within on-target region	
chr1	82,645,000	82,650,000	2	11.13	Possible Cas9 off-target cut	chr1:82,650,065-82,650,104
chr1	99,245,000	99,250,000	2	8.45	Possible Cas9 off-target cut	chr1:99,251,232-99,251,271
chr1	104,010,000	104,015,000	3	23.70	crRNA_Cas9_CS3 off-target (5MM)	chr1:104,012,371
chr1	125,180,000	125,185,000	3	66.59	Pericentromeric region	
chr1	143,215,000	143,220,000	10	90.88	Simple repeats (ATCGAATGGA)n	chr1:143,215,871-143,215,915, chr1:143,216,339-143,216,383 and chr1:143,218,115-143,218,159
chr1	150,995,000	151,000,000	2	9.63	crRNA_Cas9_CS4 off-target (3MM)	chr1:150,997,189
chr1	218,005,000	218,010,000	2	9.89	crRNA_Cas9_CS1 off-target (4MM and 1 RNA bulge)	chr1:218,004,058
chr2	115,685,000	115,690,000	7	18.20	Possible Cas9 off-target cut	chr2:115,691,125-115,691,164
chr2	188,440,000	188,445,000	7	26.22	crRNA_Cas9_CS4 off-target (5MM)	chr2:188,441,248
chr2	199,975,000	199,980,000	2	9.89	Possible Cas9 off-target cut	chr2:199,973,030-199,973,069
chr2	226,675,000	226,680,000	7	50.88	Possible Cas9 off-target cut	chr2:226,674,741-226,674,780
chr3	7,450,000	7,455,000	6	22.12	crRNA_Cas9_CS3 off-target (3MM and 2 RNA bulges)	chr3:7,457,824
chr3	50,370,000	50,375,000	7	21.13	crRNA_Cas9_CS6 off-target (4MM)	chr3:50,371,373
chr3	68,965,000	68,970,000	8	22.54	crRNA_Cas9_CS2 off-target (4MM)	chr3:68,967,418
chr3	88,620,000	88,625,000	2	10.48	Possible Cas9 off-target cut	chr3:88,620,121-88,620,160
chr3	93,470,000	93,475,000	12	66.40	Satellite (ALR/Alpha)	chr3:93,470,796-93,499,999
chr3	100,825,000	100,830,000	2	10.62	crRNA_Cas9_CS1 off-target (5MM)	chr3:100,832,818
chr4	33,465,000	33,470,000	7	44.07	crRNA_Cas9_CS1 off-target (4MM)	chr4:33,468,789
chr4	34,425,000	34,430,000	2	12.80	crRNA_Cas9_CS2 off-target (5MM)	chr4:34,427,510
chr4	49,710,000	49,715,000	6	15.80	Satellite (ALR/Alpha)	chr4:49,709,018-49,711,969
chr4	77,060,000	77,065,000	5	15.03	crRNA_Cas9_CS1 off-target (5MM)	chr4:77,066,161
chr4	119,305,000	119,310,000	2	8.66	crRNA_Cas9_CS4 off-target (3MM and 1 RNA bulge)	chr4:119,301,608
chr4	128,525,000	128,530,000	4	11.71	crRNA_Cas9_CS6 off-target (3MM and 1 RNA bulge)	chr4:128,529,790
chr4	138,830,000	138,835,000	5	13.81	crRNA_Cas9_CS6 off-target (3MM and 1 RNA bulge)	chr4:138,835,429
chr4	169,820,000	169,825,000	2	30.24	Simple repeats: (AGATGA)n, (AGATGA)n and (ATGAAG)n	chr4:169,821,398-169,821,555, chr4:169,821,166-169,821,207 and chr4:169,821,604-169,821,782
chr4	175,195,000	175,200,000	4	25.47	crRNA_Cas9_CS1 off-target (5MM)	chr4:175,194,784
chr5	34,185,000	34,190,000	2	11.81	LINEs, SINEs and LTR	chr5:34,178,830-34,197,127
chr7	31,075,000	31,080,000	3	12.42	Possible Cas9 off-target cut	chr7:31,083,397-31,083,436
chr7	56,370,000	56,375,000	4	11.59		
chr7	90,465,000	90,470,000	7	42.64	crRNA_Cas9_CS4 off-target (5MM)	chr7:90,464,125
chr8	72,890,000	72,895,000	3	15.24	crRNA_Cas9_CS1 off-target (4MM)	chr8:72,891,535
chr9	63,820,000	63,825,000	2	9.72	Simple repeat (CA)n	chr9:63,823,016-63,823,908
chr10	81,885,000	81,890,000	3	9.69	Possible Cas9 off-target cut	chr10:81,888,922-81,888,961
chr10	85,675,000	85,680,000	3	11.00	crRNA_Cas9_CS6 off-target (3MM)	chr10:85,680,865
chr11	18,280,000	18,285,000	2	9.28	Possible Cas9 off-target cut	chr11:18,281,823-18,281,862
chr11	102,330,000	102,335,000	7	31.51	crRNA_Cas9_CS6 off-target (3MM)	chr11:102,332,759
chr11	120,870,000	120,875,000	9	60.65	crRNA_Cas9_CS3 off-target (4MM)	chr11:120,871,634
chr12	510,000	515,000	2	20.74	Simple repeat (ACCTTCC)n	chr12:511,220-513,215
chr12	1,355,000	1,360,000	3	10.58	crRNA_Cas9_CS4 off-target (4MM and 2 DNA bulges)	chr12:1,358,769
chr12	33,005,000	33,010,000	6	34.15	crRNA_Cas9_CS1 off-target (2MM)	chr12:33,001,559
chr12	40,215,000	40,220,000	2	16.01	crRNA_Cas9_CS5 off-target (5MM)	chr12:40,213,906
chr12	46,905,000	46,910,000	4	17.24	Possible Cas9 off-target cut	chr12:46,903,902-46,903,941
chr12	112,475,000	112,480,000	7	29.03	crRNA_Cas9_CS6 off-target (5MM)	chr12:112,481,150
chr13	84,895,000	84,900,000	2	9.52	Possible Cas9 off-target cut	chr13:84,896,682-84,896,721
chr13	106,945,000	106,950,000	6	28.75	crRNA_Cas9_CS6 off-target (4MM)	chr13:106,946,989
chr15	20,340,000	20,345,000	2	10.84	Simple repeat (AGCCAGCCA)n	chr15:20,340,242-20,344,102
chr15	40,910,000	40,915,000	2	13.95	crRNA_Cas9_CS3 off-target (4MM)	chr15:40,913,887
chr16	10,675,000	10,680,000	2	10.66	Possible Cas9 off-target cut	chr16:10,683,998-10,684,037
chr16	46,385,000	46,390,000	11	181.73	Simple repeats	chr16:46,382,586-46,404,778
chr17	14,140,000	14,145,000	7	44.20	crRNA_Cas9_CS4 off-target (3MM)	chr17:14,148,063
chr17	75,660,000	75,665,000	7	42.15	crRNA_Cas9_CS4 off-target (4MM)	chr17:75,658,673
chr18	47,075,000	47,080,000	2	9.03	crRNA_Cas9_CS4 off-target (3MM)	chr18:47,081,745
chr18	56,455,000	56,460,000	2	12.29	crRNA_Cas9_CS3 off-target (3MM and 1 RNA bulge)	chr18:56,458,791
chr20	7,635,000	7,640,000	11	50.95	Possible Cas9 off-target cut	chr20:7,639,589-7,639,628
chr20	15,310,000	15,315,000	4	12.12	crRNA_Cas9_CS6 off-target (3MM and 1 RNA bulge)	chr20:15,311,661
chr20	29,085,000	29,090,000	2	8.46	LINE (L1M4)	chr20:29,089,404-29,090,010
chr21	5,235,000	5,240,000	2	8.82	LTR (THE1C)	chr21:5,235,998-5,236,375
chr21	8,220,000	8,225,000	2	19.76	Simple repeats: (TC)n and (TCTC)n	chr21:8,219,986-8,220,189 and chr21:8,232,546-8,235,312
chr21	8,430,000	8,435,000	6	36.73	LINEs, SINEs and simple repeats	chr21:8,391,218-8,472,701
chr21	10,330,000	10,335,000	2	9.85	LINEs, SINEs and simple repeats	chr21:10,324,679-10,349,988
chr21	10,360,000	10,365,000	2	9.97	LINEs, SINEs and simple repeats	chr21:10,355,291-10,376,867
chr21	10,425,000	10,430,000	2	11.20	Simple repeat (GTGGTG)n	chr21:10,426,491-10,427,038
chr22	11,210,000	11,215,000	4	36.72	Satellite DNA (SAR)	chr22:11211026-11211797
chr22	18,890,000	18,895,000	5	28.99	Satellite DNAs (HSATI)	chr22:18,890,645-18,891,207, chr22:18,892,654-18,893,219, chr22:18,894,119-18,894,685 and chr22:18,896,419-18,896,985
chrY	11,310,000	11,315,000	4	16.62	Satellite (BSR/Beta)	chrY:11,308,883-11,314,125
chrY	20,780,000	20,785,000	4	20.20	crRNA_Cas9_CS4 off-target (4MM)	chrY:20,779,939
chrY	56,830,000	56,835,000	4	15.70	Subtelomeric region	

Suppl. Table 6 Off-target 5,000-bp windows found enriched across the genome in two or more of the nanopore sequenced samples. Off-target enrichment threshold was set at 8 per sample as was the minimum mean depth of coverage seen in the on-target region of the nanopore sequenced samples. Sample SPG001 was not considered for the off-target analysis due to its high mean genome-wide depth of coverage (15.77x).

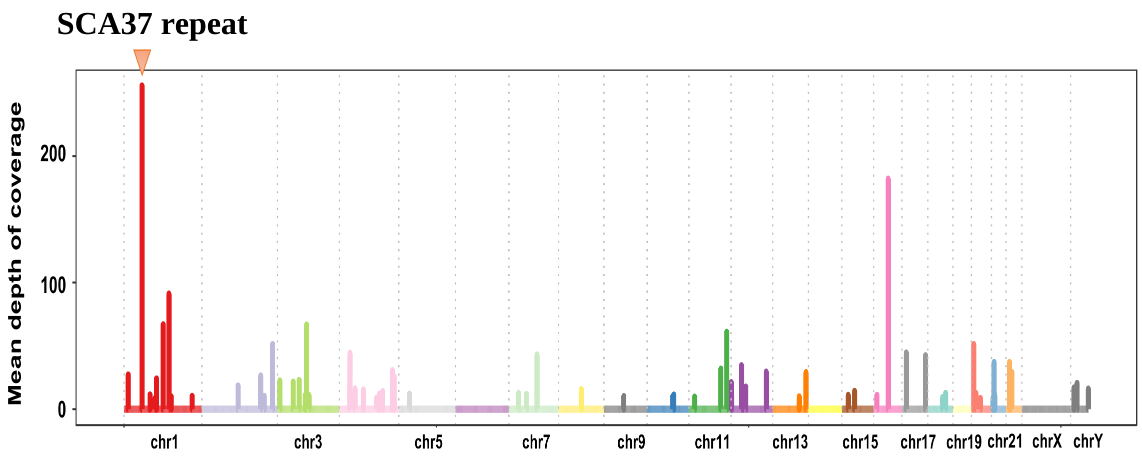

Suppl. Figure 3 Representation of the 68 off-target 5,000-bp windows found enriched across the genome as the mean depth of coverage in the enriched samples. On-target region is marked with an orange triangle.

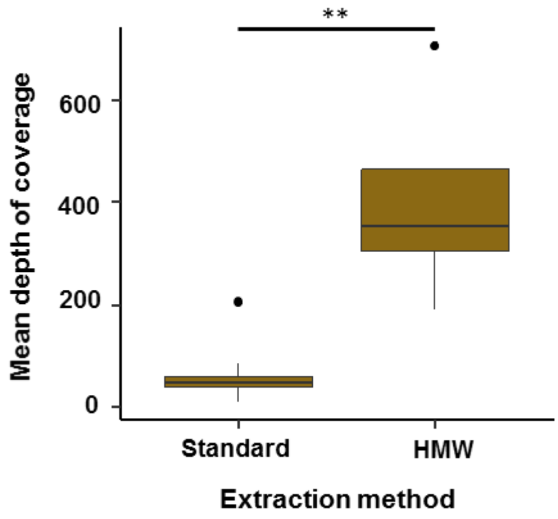

Suppl. Figure 4 Higher on-target mean depth of coverage for HMW gDNA extracted samples compared to the ones extracted with Chemagen Magnetic Separation Module DNA (p = 0.0003 n = 14). Mean depth of coverage within the targeted region was 4.4x higher in HMW gDNA extracted samples. Extraction methods were not compared using the same samples.

Fast/Hac/Sup comparison (n=2)

Sample ID	Number of CRISPR/Cas9-target region reads	Fast basecalling mode				Hac basecalling mode				Sup basecalling mode			
		Mismatch Rate	Deletion Rate	Insertion Rate	Error Rate (%)	Mismatch Rate	Deletion Rate	Insertion Rate	Error Rate (%)	Mismatch Rate	Deletion Rate	Insertion Rate	Error Rate (%)
**SPA002-PBL**	**196**	0.019	0.013	0.010	4.160%	0.019	0.014	0.009	4.230%	0.018	0.012	0.008	3.866%
**SPB001-PBL**	**22**	0.024	0.017	0.011	5.169%	0.021	0.016	0.010	4.614%	0.023	0.014	0.010	4.616%
**mean**		0.021	0.015	0.010	**4.665%**	0.020	0.015	0.010	**4.422%**	0.020	0.013	0.009	**4.241%**
**SD**		0.004	0.002	0.001	**0.713%**	0.001	0.001	0.000	**0.272%**	0.003	0.001	0.001	**0.531%**

Fast/Sup comparison (n=8)

Sample ID	Number of CRISPR/Cas9-target region reads	Fast basecalling mode				Sup basecalling mode			
		Mismatch Rate	Deletion Rate	Insertion Rate	Error Rate (%)	Mismatch Rate	Deletion Rate	Insertion Rate	Error Rate (%)
**SPA001-PBL**	**1281**	0.023	0.020	0.012	5.537%	0.014	0.010	0.007	3.093%
**SPA002-PBL**	**196**	0.019	0.013	0.010	4.160%	0.018	0.012	0.008	3.866%
**SPB001-PBL**	**22**	0.024	0.017	0.011	5.169%	0.023	0.014	0.010	4.616%
**SPC001-PBL**	**336**	0.022	0.022	0.012	5.570%	0.013	0.011	0.006	3.010%
**SPD001-PBL**	**315**	0.023	0.024	0.012	5.807%	0.013	0.012	0.006	3.205%
**SPE001-PBL**	**462**	0.028	0.020	0.018	6.560%	0.016	0.011	0.009	3.587%
**SPF001-PBL**	**397**	0.030	0.022	0.017	6.786%	0.019	0.012	0.009	4.053%
**SPG001-PBL**	**495**	0.029	0.021	0.019	6.856%	0.015	0.010	0.009	3.375%
**mean**		0.025	0.020	0.014	**5.806%**	0.016	0.012	0.008	**3.601%**
**SD**		0.004	0.003	0.003	**0.916%**	0.003	0.001	0.001	**0.550%**

Hac/Sup comparison (n=5)

Sample ID	Number of CRISPR/Cas9-target region reads	Hac basecalling mode				Sup basecalling mode			
		Mismatch Rate	Deletion Rate	Insertion Rate	Error Rate (%)	Mismatch Rate	Deletion Rate	Insertion Rate	Error Rate (%)
**SPA002-PBL**	**196**	0.019	0.014	0.009	4.230%	0.018	0.012	0.008	3.866%
**SPA003-SF**	**2168**	0.017	0.012	0.009	3.787%	0.016	0.010	0.008	3.355%
**SPB001-PBL**	**22**	0.021	0.016	0.010	4.614%	0.023	0.014	0.010	4.616%
**SPC001-SF**	**3521**	0.018	0.012	0.009	3.941%	0.016	0.011	0.008	3.465%
**SPH001-PBL**	**499**	0.021	0.015	0.013	4.870%	0.019	0.013	0.010	4.182%
**mean**		0.019	0.014	0.010	**4.289%**	0.018	0.012	0.009	**3.897%**
**SD**		0.002	0.001	0.001	**0.453%**	0.003	0.001	0.001	**0.520%**

Suppl. Table 7 Error rate of nanopore sequencing comparing Fast, Hac and Sup Guppy basecalling modes. PBL samples SPA001 and SPB001 were basecalled with all three basecalling modes. Error rates of nanopore sequenced samples basecalled with Sup mode was assessed considering all 14 nanopore sequenced samples, PBL samples SPA001, SPA002, SPB001, SPC001, SPD001, SPE001, SPF001 and SPG001 and samples SPA002-PBL, SPA003-SF, SPB001-PBL, SPC001-SF and SPH001-PBL had been previously basecalled with Fast and Hac modes, respectively.

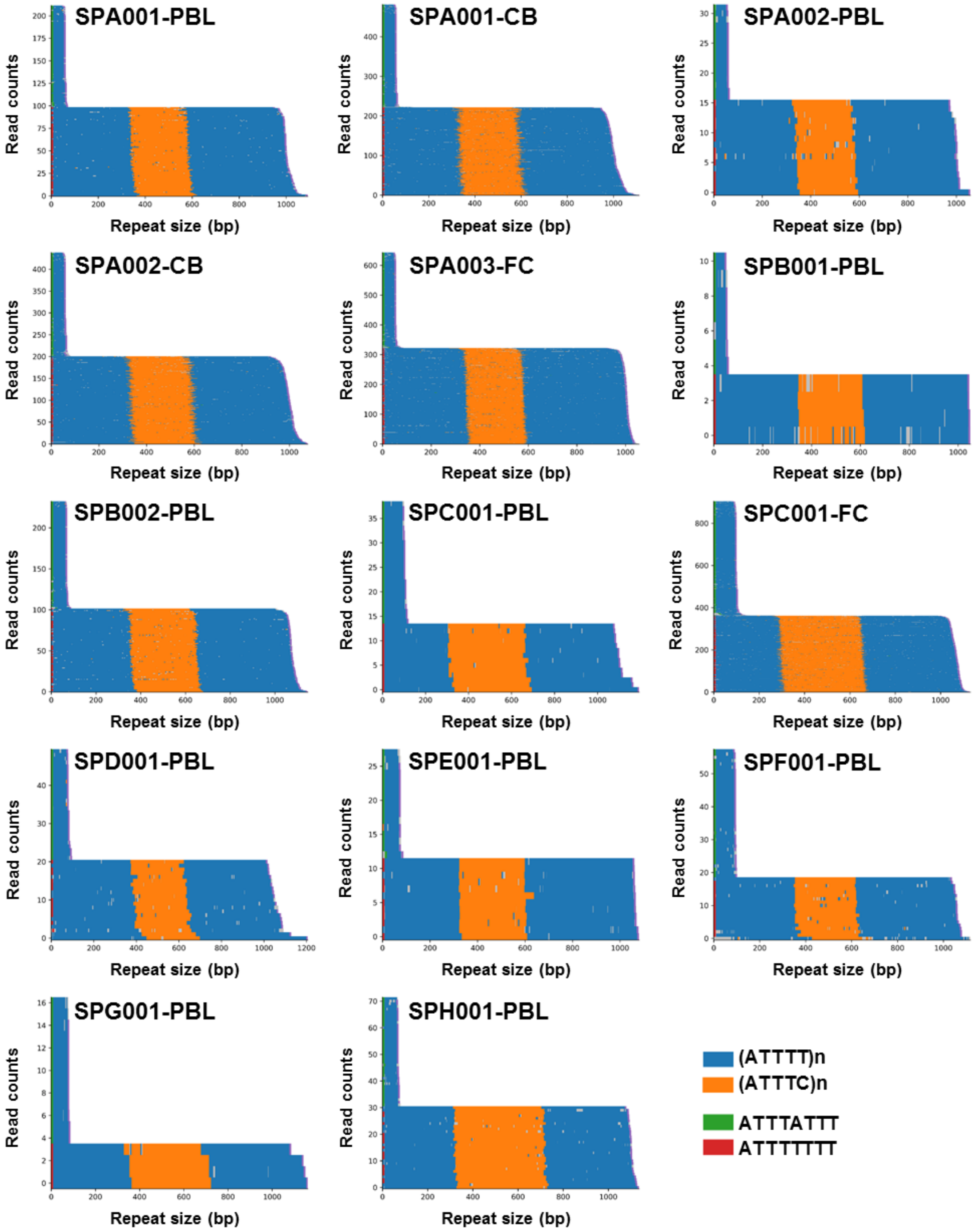

Suppl. Figure 5 Waterfall plots showing a pure WT-(ATTTT)n allele and a SAC37 5'-(ATTTT)n-(ATTTC)n-(ATTTT)n-3' pathogenic repeat tract for all sequenced samples. The flanking 5' sequence in WT alleles differed from the 5' sequence flanking SCA37 alleles (ATTTATTT and ATTTTTTT, respectively). Waterfall plots generated using the SUP basecalling guppy mode with filtered reads in which the WT-(ATTTT)n repeat number value differed >40% of its median value for that sample or >20% for the SCA37 pathogenic repeats.

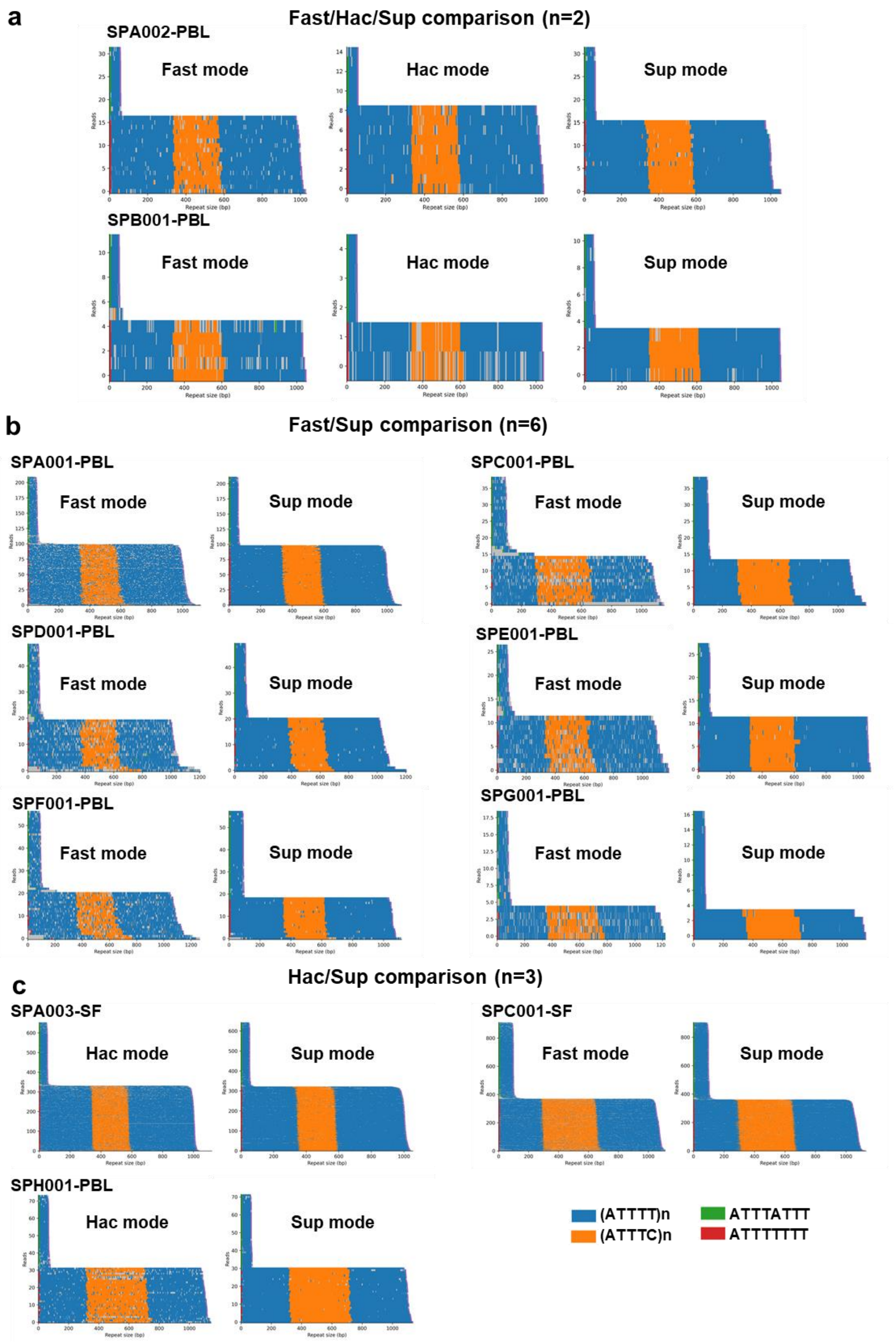

Suppl. Figure 6 Waterfall plots showing the repeat size, configuration and composition of the ATTTT/ATTTC *DAB1* repeat expansion for the Fast, Hac and SUPGuppy basecalling modes (**a**) and for Fast (left) and the Sup (right) Guppy modes (**b**) or Hac (left) and Sup (right) Guppy modes (**c**). Comparison of waterfall plots showed higher number of "other" sequences (in grey) within the repeat sequence in the samples basecalled with fast or hac mode that represented error sequences due to the basecalling process and decreased in number on sequences generated with the SUP mode.

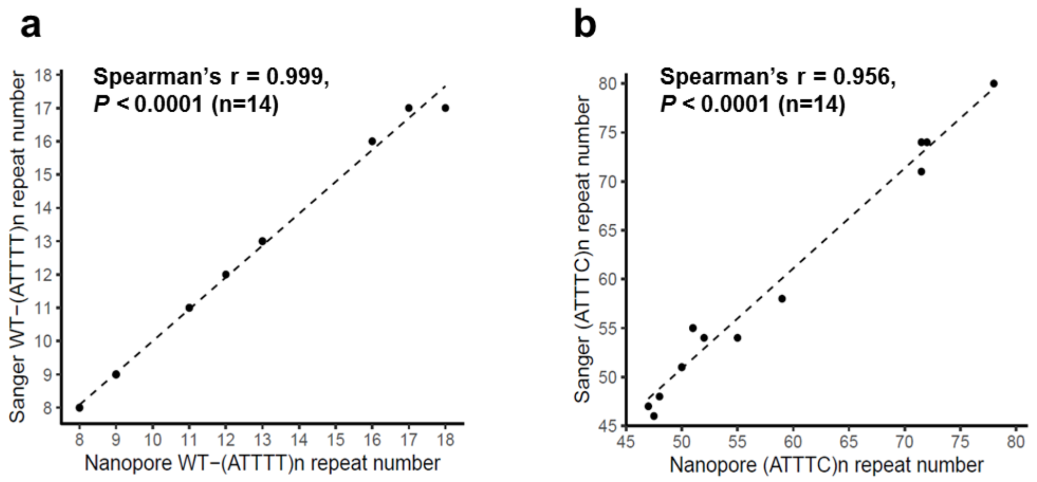

Suppl. Figure 7 (**a**) Significant correlation was observed between Sanger and nanopore sequencing for the WT-(ATTTT)n repeat allele (r=0.999, P<0.0001 (n=14)); (**b**) and for the SCA37 (ATTTC)n repeat allele (r=0.956, P < 0.0001 (n=14)).

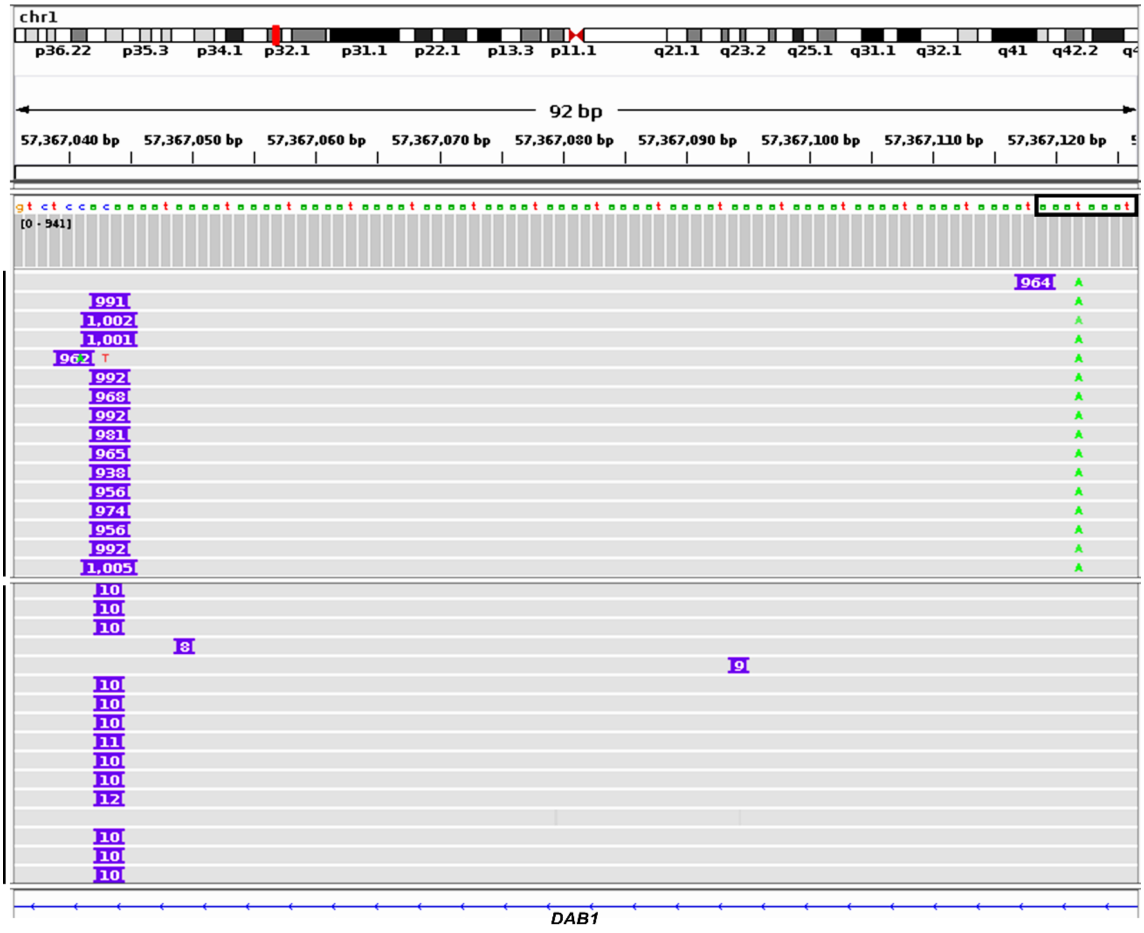

Suppl. Figure 8 IGV snapshot showing in genomic sense the reads with the ATTTTTTT sequence preceding the pathogenic SCA37 repeat (top) in contrast with the ATTTATTT sequence preceding the WT-(ATTTT)n repeat (bottom) for the SF sample SPC001.

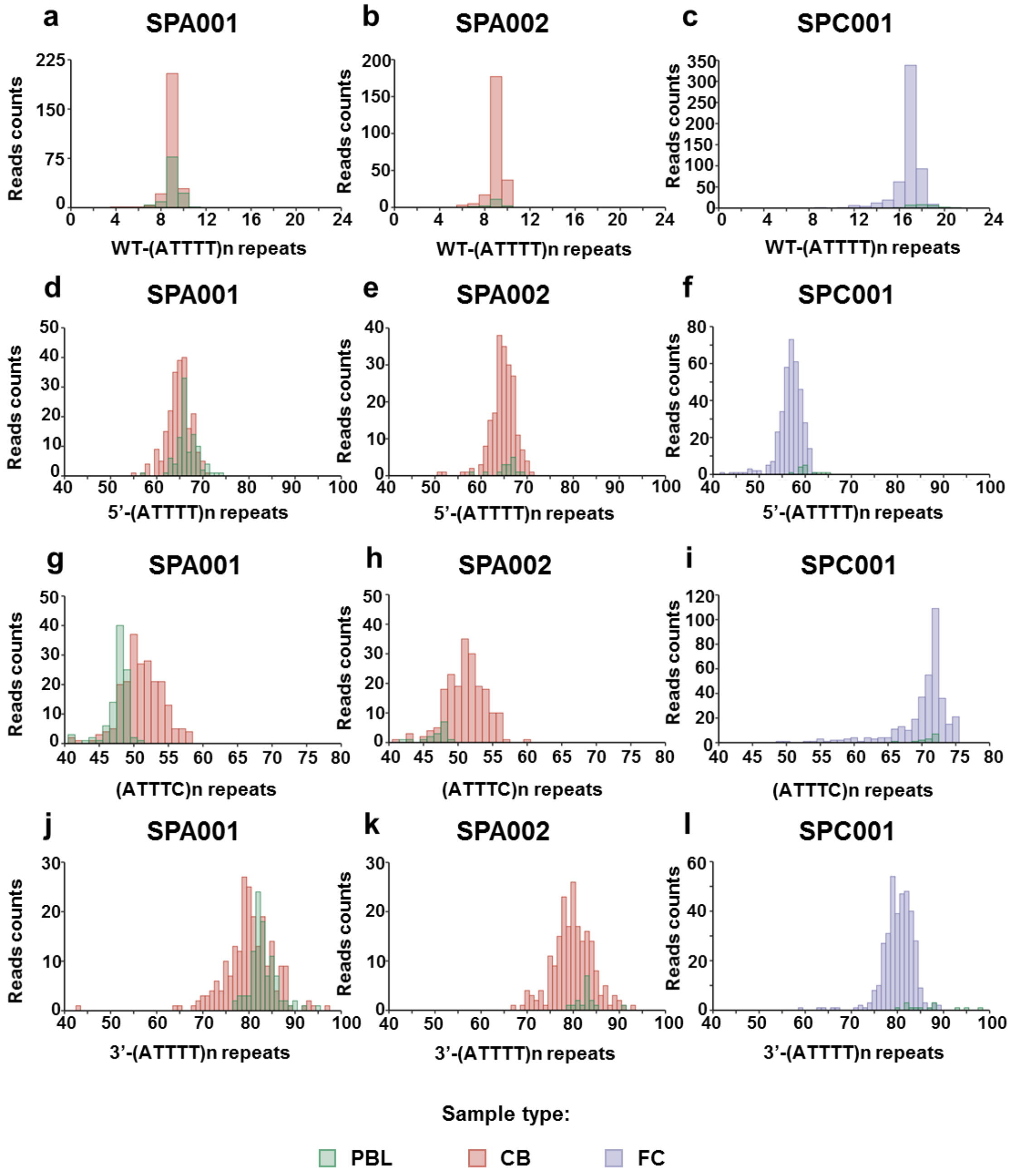

Suppl. Figure 9 Histograms showing read count peaks for WT-(ATTTT) and SCA37 5' -(ATTTT)-(ATTTC)-3'-(ATTTT) repeats alleles by sample type. A peak exclusion threshold of one or less contiguous reads in the tail was used for repeat instability calculation.

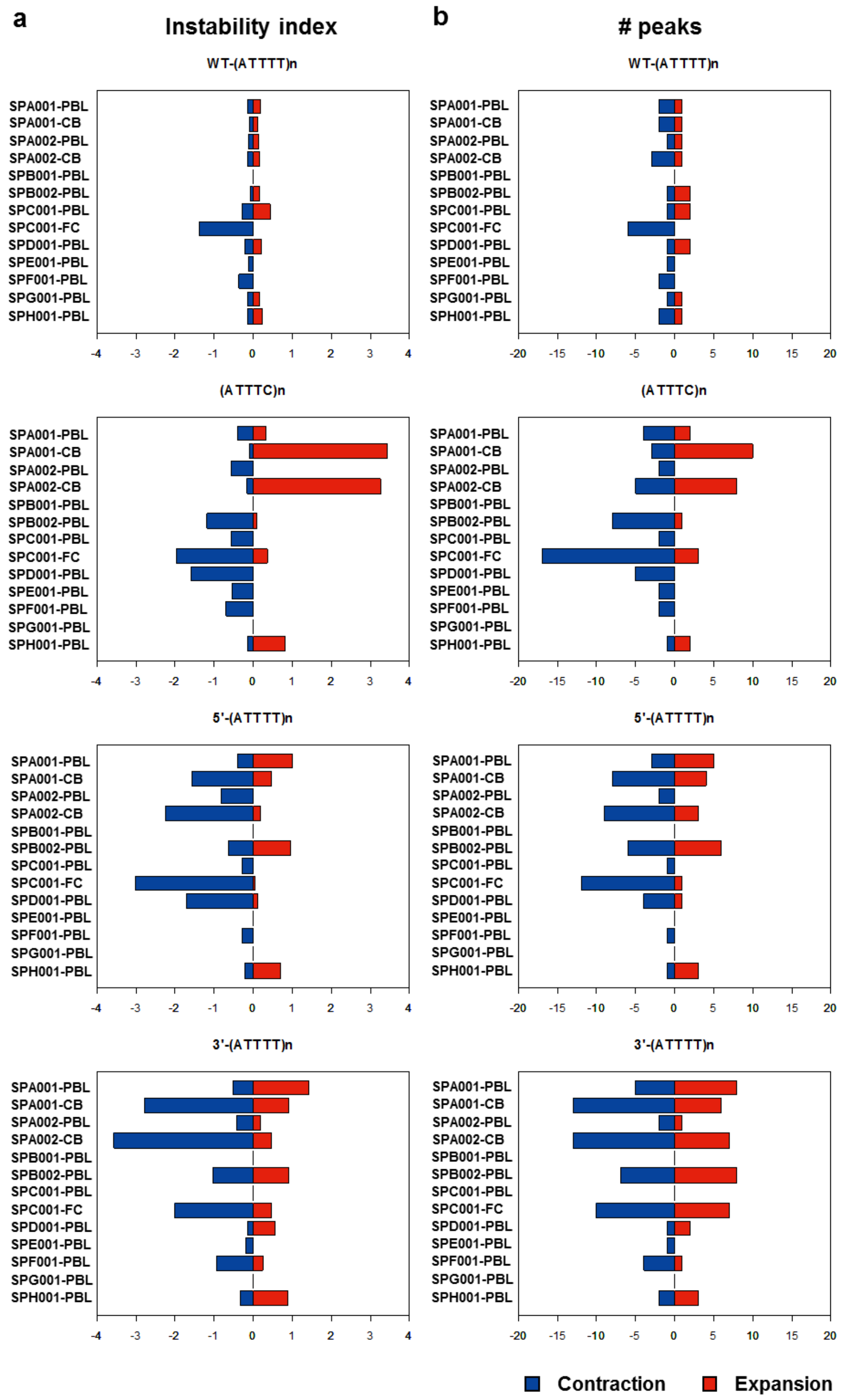

Suppl. Figure 10 Absolute contraction and expansion instability indexes (**a**) and number of contracted/expanded peaks (**b**) determined for all peripheral blood lymphocyte (PBL) samples and their relative instability indexes from cerebellar (CB) or fibroblasts (FC) samples

Motif	Type of sample	Sample ID	Instability index	Contraction index	Expansion index
ATTTT	Blood	SPA001	0.04	-0.15	0.20
		SPA002	0.00	-0.13	0.13
	Cerebellum	SPA001	0.01	-0.11	0.11
		SPA002	0.00	-0.15	0.15
	Blood	SPC001	0.13	-0.30	0.43
	Fibroblasts	SPC001	-1.40	-1.40	0.00
SCA37ATTTC	Blood	SPA001	-0.09	-0.41	0.32
		SPA002	-0.58	-0.58	0.00
	Cerebellum	SPA001	3.33	-0.10	3.43
		SPA002	3.09	-0.17	3.26
	Blood	SPC001	-0.58	-0.58	0.00
	Fibroblasts	SPC001	-1.62	-1.98	0.36
SCA37 3'(ATTTT)n	Blood	SPA001	0.92	-0.51	1.42
		SPA002	-0.25	-0.42	0.17
	Cerebellum	SPA001	-1.89	-2.80	0.92
		SPA002	-3.10	-3.58	0.48
	Blood	SPC001	0.00	0.00	0.00
	Fibroblasts	SPC001	-1.53	-2.01	0.48
SCA37 5'(ATTTT)n	Blood	SPA001	0.59	-0.41	1.00
		SPA002	-0.82	-0.82	0.00
	Cerebellum	SPA001	-1.11	-1.58	0.47
		SPA002	-2.07	-2.25	0.19
	Blood	SPC001	-0.29	-0.29	0.00
	Fibroblasts	SPC001	-3.07	-3.03	0.04

Suppl. Table 8 Absolute contraction and expansion instability indexes and number of contracted/expanded peaks determined for all peripheral blood lymphocyte (PBL) samples and their relative instability indexes from cerebellar (CB) or fibroblasts (FC) samples.

ID SNP	Chromosomic localization (chr1, hg38)	Change	SPA001		SPA002		SPA003	SPB001	SPB002	SPC001		SPD001	SPE001	SPF001	SPG001	SPH001	Nucleotide in expanded allele	Nucleotide in expanded allele (Ref/Alt)
			PBL	CB	PBL	CB	SF	PBL	PBL	PBL	SF	PBL	PBL	PBL	PBL	PBL		
rs11588755	57353532	g.57353532G>A	A/A	A/A	-	A/A	A/A	-	A/A	A/A	A/A	A/A	G/A	A/A	G/A	A/A	**A**	Alt
rs11590708	57353595	g.57353595T>G	G/G	G/G	-	G/G	G/G	-	G/G	G/G	G/G	G/G	T/G	G/G	T/G	G/G	**G**	Alt
rs12240237	57354148	g.57354148T>A	A/A	A/A	A/A	A/A	A/A	A/A	A/A	A/A	A/A	A/A	T/A	A/A	T/A	A/A	**A**	Alt
rs12741210	57354654	g.57354654G>A	A/A	A/A	A/A	A/A	A/A	A/A	A/A	A/A	A/A	A/A	G/A	A/A	G/A	A/A	**A**	Alt
rs17423255	57354694	g.57354694A>C	C/C	C/C	C/C	C/C	C/C	C/C	C/C	C/C	C/C	C/C	A/C	C/C	A/C	C/C	**C**	Alt
rs1424465	57354867	g.57354867C>T	T/T	T/T	T/T	T/T	T/T	T/T	T/T	T/T	T/T	T/T	C/T	T/T	C/T	T/T	**T**	Alt
rs10493217	57354965	g.57354965C>T	C/C	C/C	C/C	C/C	C/T	C/C	C/C	C/C	C/C	C/C	C/C	C/C	C/C	C/C	**C**	Ref
rs1424464	57355005	g.57355005G>A	A/A	A/A	A/A	A/A	A/A	A/A	A/A	A/A	A/A	A/A	G/A	A/A	G/A	A/A	**A**	Alt
rs1424463	57355025	g.57355025A>C	C/C	C/C	C/C	C/C	C/C	C/C	C/C	C/C	C/C	C/C	A/C	C/C	A/C	C/C	**C**	Alt
rs75751307	57356215	g.57356215C>A	C/C	C/C	C/C	C/C	C/A	C/C	C/C	C/C	C/C	C/C	C/C	C/C	C/C	C/C	**C**	Ref
rs116140178	57356350	g.57356350C>T	C/C	C/C	C/C	C/C	C/T	C/C	C/C	C/C	C/C	C/C	C/C	C/C	C/C	C/C	**C**	Ref
rs1834151	57357038	g.57357038C>T	T/T	T/T	T/T	T/T	T/T	T/T	T/T	T/T	T/T	T/T	C/T	T/T	C/T	T/T	**T**	Alt
rs1424462	57357080	g.57357080T>C	C/C	C/C	C/C	C/C	C/C	C/C	C/C	C/C	C/C	C/C	T/C	C/C	T/C	C/C	**C**	Alt
rs61769669	57357310	g.57357310A>C	A/C	A/C	A/C	A/C	A/C	A/C	A/C	A/C	A/C	A/C	A/C	A/C	A/C	A/C	**C**	Alt
rs61769670	57357360	g.57357360G>C	C/C	C/C	C/C	C/C	G/C	C/C	C/C	C/C	C/C	C/C	G/C	C/C	G/C	C/C	**C**	Alt
rs11207017	57357891	g.57357891G>T	T/T	T/T	T/T	T/T	T/T	T/T	T/T	T/T	T/T	T/T	G/T	T/T	G/T	T/T	**T**	Alt
rs12565446	57358243	g.57358243C>A	A/A	A/A	A/A	A/A	A/A	A/A	A/A	A/A	A/A	A/A	C/A	A/A	C/A	A/A	**A**	Alt
rs12565636	57358279	g.57358279C>T	T/T	T/T	T/T	T/T	T/T	T/T	T/T	T/T	T/T	T/T	C/T	T/T	C/T	T/T	**T**	Alt
rs2113454	57359677	g.57359677G>A	A/A	A/A	A/A	A/A	G/A	A/A	A/A	A/A	A/A	A/A	G/A	A/A	G/A	A/A	**A**	Alt
rs114879618	57359819	g.57359819T>C	T/T	T/T	T/T	T/T	T/T	T/T	T/C	T/T	T/T	T/T	T/T	T/T	T/T	T/T	**T**	Ref
rs10889038	57361094	g.57361094T>C	C/C	C/C	C/C	C/C	C/C	C/C	C/C	C/C	C/C	C/C	T/C	C/C	T/C	C/C	**C**	Alt
rs78141582	57361845	g.57361845A>G	A/A	A/A	A/A	A/A	A/G	A/A	A/G	A/A	A/A	A/A	A/A	A/A	A/A	A/A	**A**	Ref
rs11207018	57362761	g.57362761C>T	T/T	T/T	T/T	T/T	T/T	T/T	T/T	T/T	T/T	T/T	T/T	T/T	C/T	T/T	**T**	Alt
rs6665166	57362808	g.57362808C>T	T/T	T/T	T/T	T/T	T/T	T/T	T/T	T/T	T/T	T/T	C/T	T/T	C/T	T/T	**T**	Alt
rs75203772	57362823	g.57362823A>T	A/T	A/T	A/T	A/T	A/A	A/T	A/A	A/A	A/A	A/A	A/A	A/A	A/A	A/A	**A**	Ref
rs10889039	57362873	g.57362873C>T	T/T	T/T	T/T	T/T	T/T	T/T	T/T	T/T	T/T	T/T	C/T	T/T	C/T	T/T	**T**	Alt
rs75173975	57363422	g.57363422G>A	G/G	G/G	G/G	G/G	G/G	G/G	G/G	G/G	G/G	G/G	G/G	G/G	G/G	G/A	**G**	Ref
rs72674872	57363537	g.57363537A>T	A/T	A/T	A/T	A/T	A/T	A/T	A/T	T/T	T/T	T/T	A/T	T/T	A/T	A/T	**T**	Alt
rs115844296	57363572	g.57363572T>G	T/T	T/T	T/T	T/T	T/T	T/T	T/T	T/T	T/T	T/T	T/T	T/T	T/T	T/G	**T**	Ref
rs10889040	57365338	g.57365338T>C	C/C	C/C	C/C	C/C	C/C	T/C	C/C	C/C	C/C	C/C	T/C	C/C	T/C	C/C	**C**	Alt
rs6669975	57365576	g.57365576T>C	T/C	T/C	T/C	T/C	C/C	T/C	C/C	C/C	C/C	C/C	T/C	C/C	T/C	T/C	**C**	Alt
rs7513076	57365852	g.57365852G>C	G/C	G/C	G/C	G/C	G/C	G/C	C/C	C/C	C/C	C/C	G/C	C/C	G/C	G/C	**C**	Alt
rs7535763	57366182	g.57366182A>G	A/G	A/G	A/G	A/G	G/G	A/G	G/G	G/G	G/G	G/G	A/G	G/G	A/G	A/G	**G**	Alt
rs80205519	57366241	g.57366241G>A	G/G	G/G	G/G	G/G	G/G	G/G	G/G	G/G	G/G	G/G	G/A	G/G	G/G	G/G	**G**	Ref
(ATTTC)n (Chr1:57,367,044-57,367,118)																		
rs2113453	57368017	g.57368017T>C	C/C	C/C	C/C	C/C	C/C	C/C	C/C	C/C	C/C	C/C	T/C	C/C	T/C	C/C	**C**	Alt
rs12566690	57368196	g.57368196C>T	C/T	C/T	C/T	C/T	C/T	C/T	C/T	T/T	T/T	T/T	C/T	T/T	C/T	C/T	**T**	Alt
rs11207020	57368363	g.57368363C>T	C/T	C/T	C/T	C/T	C/T	C/T	T/T	T/T	T/T	T/T	C/T	T/T	C/T	C/T	**T**	Alt
rs12137540	57369511	g.57369511C>T	C/T	C/T	C/T	C/T	C/T	C/T	T/T	T/T	T/T	T/T	C/T	T/T	C/T	C/T	**T**	Alt
rs7548078	57369841	g.57369841A>G	A/A	A/A	A/A	A/A	A/A	A/A	A/A	A/A	A/A	A/A	A/A	A/A	A/A	A/G	**A**	Ref
rs77711330	57370210	g.57370210C>T	C/C	C/C	C/C	C/C	C/C	C/C	C/T	C/C	C/C	C/C	C/C	C/C	C/C	C/C	**C**	Ref
rs7528735	57370527	g.57370527G>A	G/A	G/A	G/A	G/A	G/A	G/A	A/A	A/A	A/A	A/A	G/A	A/A	G/A	G/A	**A**	Alt
rs116382271	57370798	g.57370798C>A	C/C	C/C	C/C	C/C	C/C	C/C	C/A	C/C	C/C	C/C	C/C	C/C	C/C	C/C	**C**	Ref
rs7540817	57370816	g.57370816T>C	T/C	T/C	T/C	T/C	T/C	T/C	C/C	C/C	C/C	C/C	T/C	C/C	T/C	T/C	**C**	Alt
rs2405696	57371012	g.57371012G>A	G/A	G/A	G/A	G/A	A/A	G/A	A/A	A/A	A/A	A/A	G/A	A/A	G/A	G/A	**A**	Alt
rs7541150	57371215	g.57371215T>G	T/G	T/G	T/G	T/G	T/T	T/G	T/T	T/T	T/T	T/T	T/T	T/T	T/T	T/T	**T**	Ref
rs10047073	57375959	g.57375959G>C	G/C	G/C	G/C	G/C	C/C	G/C	C/C	C/C	C/C	C/C	G/C	C/C	G/C	-	**C**	Alt
rs10047071	57375990	g.57375990C>T	C/T	C/T	C/T	C/T	T/T	C/T	T/T	T/T	T/T	T/T	C/T	T/T	C/T	-	**T**	Alt
rs11585284	57376773	g.57376773C>T	C/T	C/T	-	C/T	C/T	C/T	T/T	T/T	T/T	-	C/T	-	C/T	-	**T**	Alt
rs6690017	57377057	g.57377057T>G	T/G	T/G	-	T/G	G/G	T/G	G/G	G/G	G/G	-	T/G	-	T/G	-	**G**	Alt
rs183761182	57381473	g.57381473T>C	T/T	T/T	-	T/T	T/T	-	T/T	T/C	T/C	-	-	-	-	-	**T**	Ref
rs17423972	57378247	g.57378247A>C	A/C	A/C	-	A/C	A/A	A/A	A/A	A/A	A/A	-	A/A	-	A/A	-	**A**	Ref
rs17115683	57378593	g.57378593T>C	T/C	T/C	-	T/C	T/T	T/C	T/T	T/T	T/T	-	T/T	-	-	-	**T**	Ref
rs12127603	57382931	g.57382931A>C	A/C	A/C	-	A/C	A/C	-	C/C	-	C/C	-	-	-	-	-	**C**	Alt
rs10493219	57383013	g.57383013A>G	A/G	A/G	-	A/G	A/G	-	G/G	-	G/G	-	-	-	-	-	**G**	Alt
rs12136107	57384130	g.57384130C>G	C/G	C/G	-	C/G	C/G	-	G/G	-	G/G	-	-	-	-	-	**G**	Alt

Suppl. Table 9 Common SNPs identified in the 14 nanopore sequenced SCA37 samples used to classify the reads generated from nanopore sequencing that mapped in the on-target region, but did not contain the expanded tract in SCA37 or WT alleles. (-) Positions with less than 3-fold coverage.

Chr	Coordinate	Allele	Mean LLR	SD LLR	Number of reads	Number of outliers	*p*-value	Test	Methylation difference (SCA37 vs WT)
1	57,371,220	SCA37	-2.13	3.43	316	9	<0.001	Mann-Whitney U test	decrease
		WT	-1.23	2.89	341	22			
1	57,371,070	SCA37	-2.41	4.17	316	5	<0.001	ANOVA	decrease
		WT	-0.89	4.20	344	9			
1	57,371,011	SCA37	-5.34	3.82	316	1	<0.001	Mann-Whitney U test	decrease
		WT	-1.87	7.52	341	2			
1	57,370,676	SCA37	-2.08	4.08	315	12	<0.001	ANOVA	decrease
		WT	0.07	4.05	338	6			
1	57,370,629	SCA37	-2.78	2.09	315	15	<0.001	Mann-Whitney U test	decrease
		WT	-2.04	2.44	338	10			
1	57,368,380	SCA37	1.70	3.70	470	16	0.011	ANOVA	increase
		WT	1.11	3.41	505	20			
1	57,368,363	SCA37	-1.05	1.71	469	24	<0.001	Mann-Whitney U test	decrease
		WT	1.02	2.95	505	18			
1	57,368,270	SCA37	1.29	1.86	465	46	0.001	ANOVA	increase
		WT	0.85	2.01	504	47			
1	57,368,196	SCA37	-2.86	3.53	465	4	<0.001	Mann-Whitney U test	decrease
		WT	1.66	2.97	503	13			
1	57,367,625	SCA37	-0.92	2.82	457	10	<0.001	Mann-Whitney U test	increase
		WT	-1.72	3.34	497	4			
1	57,367,557	SCA37	1.24	2.53	456	27	0.015	ANOVA	decrease
		WT	1.67	2.71	495	20			
1	57,367,328	SCA37	0.14	2.61	452	38	<0.001	Mann-Whitney U test	decrease
		WT	0.98	3.42	497	25			
1	57,367,004	SCA37	2.28	3.48	429	15	<0.001	Mann-Whitney U test	increase
		WT	1.48	2.81	492	15			
1	57,366,956	SCA37	2.84	2.58	430	16	0.001	ANOVA	increase
		WT	2.26	2.57	492	16			
1	57,366,050	SCA37	0.09	2.14	436	29	<0.001	ANOVA	increase
		WT	-0.62	2.18	486	49			
1	57,365,870	SCA37	-0.31	3.53	435	16	<0.001	Mann-Whitney U test	increase
		WT	-1.69	4.59	483	9			
1	57,365,807	SCA37	-0.01	3.23	436	25	0.018	ANOVA	increase
		WT	-0.54	3.40	482	36			
1	57,365,681	SCA37	0.27	2.70	436	28	<0.001	Mann-Whitney U test	increase
		WT	-0.66	3.38	482	35			
1	57,364,762	SCA37	1.29	3.25	431	2	0.006	Mann-Whitney U test	increase
		WT	0.57	2.68	479	21			
1	57,364,473	SCA37	-0.53	3.00	430	26	0.015	ANOVA	increase
		WT	-1.02	2.95	476	38			
1	57,363,421	SCA37	0.70	2.00	424	9	0.001	ANOVA	increase
		WT	0.25	1.88	471	22			
1	57,363,408	SCA37	-0.95	2.94	424	62	0.003	Mann-Whitney U test	increase
		WT	-1.82	3.80	472	46			
1	57,363,389	SCA37	-2.09	4.27	424	7	<0.001	ANOVA	increase
		WT	-3.12	4.49	472	6			
1	57,363,376	SCA37	-1.25	2.07	424	22	<0.001	Mann-Whitney U test	increase
		WT	-2.10	2.42	472	10			
1	57,362,885	SCA37	-0.39	2.84	423	18	0.028	Mann-Whitney U test	increase
		WT	-0.85	3.28	474	12			
1	57,361,787	SCA37	-2.44	4.31	409	21	<0.001	Mann-Whitney U test	increase
		WT	-3.90	5.25	451	6			
1	57,361,330	SCA37	-1.64	2.99	404	25	0.003	ANOVA	increase
		WT	-2.27	3.05	445	24			
1	57,360,184	SCA37	0.68	3.40	393	13	0.004	Mann-Whitney U test	increase
		WT	-0.03	2.75	437	28			
1	57,357,890	SCA37	1.19	1.65	257	13	0.045	ANOVA	decrease
		WT	1.50	1.68	246	16			
1	57,356,872	SCA37	2.13	3.11	259	28	0.049	ANOVA	increase
		WT	1.55	3.26	251	19			

Suppl. Table 10 Significant differentially methylated CpGs between SCA37 and WT cerebellar alleles of SPA001-CB and SPA002-CB samples.

Ped.	Ped. ID	Gender	Age of onset	Disease evolution	WT-(ATTTT)n	WT total repeat lenght	SCA37 5'-(ATTTT)n	SCA37 (ATTTC)n	SCA37 3'-(ATTTT)n	SCA37 total repeat lenght
AT-901	IV:10	F	39	38	9	45	66	48	83	993
AT-901	IV:15	M	50	23	14	70	NA	48	NA	NA
AT-901	IV:19	F	56	28	7	35	NA	55	NA	NA
AT-901	IV:21	F	40	42	8	40	NA	54	NA	NA
AT-901	IV:4	M	64	15	8	40	67	47	84	996
AT-901	IV:5	F	59	15	7	35	NA	47	NA	NA
AT-901	IV:6	M	54	NA	8	40	NA	46	NA	NA
AT-901	IV:9	F	55	27	9	45	66	48	83	992
AT-901	V:9	M	42	18	15	75	NA	57	NA	NA
AT-9012	III:10	F	45	22	7	35	NA	52	NA	NA
AT-9012	III:12	M	46	18	14	70	NA	51	NA	NA
AT-9012	III:3	F	43	27	8	40	67	50	85	1036
AT-9012	III:5	M	45	15	8	40	NA	54	NA	NA
AT-9012	III:7	M	50	23	50	261	NA	49	NA	NA
AT-59	IV:3	F	35	29	7	35	NA	48	NA	NA
AT-59	IV:7	M	44	10	9	45	NA	71	NA	NA
AT-59	IV:9	F	32	19	18	90	60	72	86	1091
AT-59	V:2	F	36	15	13	65	NA	49	NA	NA
AT-59	V:3	F	25	20	13	65	NA	48	NA	NA
AT-90	III:1	F	38	21	13	65	NA	53	NA	NA
AT-90	III:6	M	46	4	13	66	76	50	81	1043
AT-90	III:8	F	37	18	13	65	NA	51	NA	NA
AT-90	IV:1	F	26	12	7	35	NA	53	NA	NA
AT-E	I:1	M	40	NA	12	60	63	55	91	1054
AT-F	I:1	F	42	11	16	80	69	52	86	1049
AT-G	I:1	F	37	10	13	65	69	72	84	1134
PO-G	PO-G11	F	35	2	NA	NA	NA	50	NA	NA
PO-G	PO-G2	F	58	10	14	NA	62	43	NA	NA
PO-G	PO-G6	F	55	20	14	NA	66	31	NA	NA
PO-G	PO-G8	F	40	14	NA	NA	NA	51	NA	NA
PO-G	PO-G9	M	45	13	25	NA	72	53	NA	NA
PO-M	PO-M1	F	30	34	NA	NA	NA	60	NA	NA
PO-M	PO-M10	F	46	6	NA	NA	NA	60	NA	NA
PO-M	PO-M11	M	30	8	7	NA	63	61	74	990
PO-M	PO-M12	F	26	15	NA	NA	NA	60	NA	NA
PO-M	PO-M13	F	29	2	51	NA	65	65	58	940
PO-M	PO-M2	M	30	40	8	NA	64	67	64	975
PO-M	PO-M3	F	18	24	14	NA	60	75	76	1055
PO-M	PO-M4	F	32	3	7	NA	65	74	78	1085
PO-M	PO-M5	F	30	27	12	NA	69	53	90	1060
PO-M	PO-M6	F	34	6	14	NA	67	62	79	1040
PO-M	PO-M7	F	40	6	NA	NA	NA	60	NA	NA
PO-M	PO-M8	M	27	30	13	NA	69	73	NA	NA
PO-M	PO-M9	F	23	38	20	NA	71	54	81	1030
PO-MS	PO-MS1	M	40	38	19	NA	73	46	81	1000
PO-MS	PO-MS2	M	47	31	19	NA	69	52	90	1055
PO-MS	PO-MS3	M	57	14	19	NA	72	46	NA	NA
PO-MS	PO-MS4	F	31	6	12	NA	67	61	NA	NA
PO-R	PO-R1	F	30	47	52	NA	72	56	73	1005
PO-R	PO-R2	F	40	29	52	NA	69	52	73	970
PO-R	PO-R3	F	31	47	14	NA	75	57	NA	NA
PO-R	PO-R4	M	55	14	NA	NA	NA	59	NA	NA
PO-R	PO-R5	F	28	12	NA	NA	NA	71	NA	NA
PO-R	PO-R6	F	30	5	NA	NA	NA	71	NA	NA
PO-R	PO-R7	M	32	5	51	NA	69	69	NA	NA
PO-R	PO-R8	F	23	54	12	NA	78	55	68	1005

Suppl. Table 11 Clinical and molecular data from 56 SCA37 patients from seven Spanish and four Portuguese kindreds.

	Age of onset	Disease evolution	Gender	WT-(ATTTT)n	WT-total repeat lenght	SCA37-5’-(ATTTT)n	(ATTTC)n	SCA37-3’-(ATTTT)n	SCA37-total repeat lenght	Country
**Age of onset**										
**Disease evolution**	-0.169 (0.199)									
**Gender**	**0.349 (0.009)**	-0.066 (0.644)								
**WT-(ATTTT)n**	-0.135 (0.335)	0.001 (0.966)	0.056 (0.694)							
**WT-total repeat lenght**	0.003 (0.971)	-0.061 (0.833)	0.260 (0.165)	**1.000 (<0.001)**						
**SCA37-5’-(ATTTT)n**	-0.004 (0.963)	0.371 (0.061)	0.179 (0.405)	0.139 (0.354)	-0.082 (0.981)					
**(ATTTC)n**	**-0.572 (<0.001)**	-0.202 (0.156)	-0.090 (0.370)	0.065 (0.640)	-0.004 (0.986)	**-0.313 (0.040)**				
**SCA37-3’-(ATTTT)n**	**0.458 (0.028)**	-0.080 (0.601)	0.147 (0.486)	**-0.445 (0.007)**	0.213 (0.991)	-0.011 (0.952)	-0.338 (0.095)			
**SCA37-total repeat lenght**	-0.078 (0.814)	-0.280 (0.093)	-0.144 (0.494)	**-0.419 (0.012)**	0.596 (0.546)	-0.150 (0.431)	**0.480 (0.017)**	**0.576 (<0.001)**		
**Country**	**-0.356 (0.007)**	0.000 (1.000)	-0.138 (0.218)	**0.355 (0.011)**	nan (1.000)	0.134 (0.471)	**0.272 (0.033)**	**-0.500 (0.014)**	-0.371 (0.072)	

Suppl. Table 12 Pearson’s correlation coefficient (r) and the associated *p*-value in brackets between “Age of onset”, “Diseases evolution”, “Gender”, “WT-(ATTTT)n”, “WT-total-repeat-length”, “SCA37-5’-(ATTTT)n”, “SCA37-(ATTTC)n”, “SCA37-3’-(ATTTT)n”, “SCA37-total-repeat-length” and “Country of origin”. Significant correlations are highlighted in bold.

References

1. Liu X, Homma A, Sayadi J, Yang S, Ohashi J, Takumi T. Sequence features associated with the cleavage efficiency of CRISPR/Cas9 system. Sci Rep. 2016 Jan 27;6:19675.
2. Thyme SB, Akhmetova L, Montague TG, Valen E, Schier AF. Internal guide RNA interactions interfere with Cas9-mediated cleavage. Nat Commun. 2016 Jun 10;7:11750.
3. Keraite I, Becker P, Canevazzi D, Frias-López C, Dabad M, Tonda-Hernandez R, et al. A method for multiplexed full-length single-molecule sequencing of the human mitochondrial genome. Nat Commun. 2022 Oct 6;13(1):5902.
4. Li B, Ren N, Yang L, Liu J, Huang Q. A qPCR method for genome editing efficiency determination and single-cell clone screening in human cells. Sci Rep. 2019 Dec 11;9(1):18877.
