# Supplementary material for: Novel genotype–phenotype correlations, differential cerebellar allele-specific methylation, and a common origin of the (ATTTC)n insertion in spinocerebellar ataxia type 37
Source: Hum Genet. 2024 Feb 23;143(3):211–32. doi: 10.1007/s00439-024-02644-7 (PMC11043136; doi:10.1007/s00439-024-02644-7)
Supplement: Supplementary file 3 — Supplementary file3 (PDF 299 KB) [file 439_2024_2644_MOESM3_ESM.pdf]

Novel genotype-phenotype correlations, differential cerebellar allele-specific methylation, and a common origin of the $(ATTTC)_n$ insertion in spinocerebellar ataxia type 37

Human Genetics

Marina Sanchez-Flores[1†], Marc Corral-Juan[1†], Esther Gasch-Navalón[1], Davide Cirillo[2], Ivelisse Sanchez[1], Antoni Matilla-Dueñas[1*].

1. Functional and Translational Neurogenetics Unit, Department of Neuroscience, Research Institute Germans Trias i Pujol (IGTP), Universitat Autònoma de Barcelona-Can Ruti Campus, Badalona, Barcelona, Spain.
2. Barcelona Supercomputing Center (BSC), Barcelona, Spain.

†Marina Sanchez-Flores and Marc Corral-Juan have contributed equally to this work.

*To whom correspondence should be addressed at:

Dr. Antoni Matilla-Dueñas

Head of the Neurogenetics Unit

Health Sciences Research Institute Germans Trias i Pujol (IGTP)

Carretera de Can Ruti, Camí de les Escoles s/n

08916 Badalona, Barcelona, Spain

Telephone: +34 930 330 532

Fax: +34 934 978 654

E-mail: amatilla@igtp.cat

SNP ID	PO-M				PO-G			PO-R			PO-MS				PO-C	PO-D	AT-901			AT-9012				AT-59		AT-90	AT-E	AT-F	AT-G	AT-H	SCA37	Population	LD analysis	
	II.12	III.15	III.16	IV.2	II.1	II.7	IV.1	II.2	II.10	III.7	MS1	MS2	MS3	MS4	C1	D1	IV:4	IV:9	V:9	III:3	III:12	IV:4	IV:9	VI:9	V:3	III:6	I:1	I:1	I:1	I:1	Cases	MAF	Chi Square	*p*-value
rs565332393	2	2	2	2	2	2	2	2	2	2	2	2	2	2	1	1	*[2]*	*2*	*2*	[1]	*[2]*	[1]	*[2]*	*[2]*	*[2]*	*[2]*	*[2]*	*[2]*	[1]	[1]	24/30	0.005	338.57	< 0.00001
rs762335464	2	2	2	2	2	2	2	2	2	2	2	2	2	2	1	1	*[2]*	*2*	*2*	[1]	*[2]*	[1]	*[2]*	*[2]*	*[2]*	*[2]*	*[2]*	*[2]*	[1]	[1]	24/30	0.009	178.70	< 0.00001
rs142969184	2	2	2	2	2	2	2	2	2	2	2	2	2	2	1	1	*[2]*	*2*	*2*	[1]	*[2]*	[1]	*[2]*	*[2]*	*[2]*	*[2]*	*[2]*	*[2]*	[1]	[1]	24/30	0.029	83.87	< 0.00001
rs761751006	2	2	2	2	2	2	2	2	2	2	2	2	2	2	1	1	*[2]*	*2*	*2*	[1]	*[2]*	[1]	*[2]*	*[2]*	*[2]*	*[2]*	*[2]*	*[2]*	[1]	[1]	24/30	0.003	499.68	< 0.00001
rs1004697722	2	2	2	2	2	2	2	2	2	2	2	2	2	2	1	1	*[2]*	*2*	*2*	[1]	*[2]*	[1]	*[2]*	*[2]*	*[2]*	*[2]*	*[2]*	*[2]*	[1]	[1]	24/30	0.001	1016.33	< 0.00001
rs777060331	2	2	2	2	2	2	2	2	2	2	2	2	2	2	1	1	*[2]*	*2*	*2*	[1]	*[2]*	[1]	*[2]*	*[2]*	*[2]*	*[2]*	*[2]*	*[2]*	[1]	[1]	24/30	0.008	211.32	< 0.00001
rs1022106103	2	2	2	2	2	2	2	2	2	2	2	2	2	2	1	1	*[2]*	2	2	[1]	*[2]*	[1]	*[2]*	*[2]*	*[2]*	*[2]*	*[2]*	*[2]*	[1]	[1]	24/30	0.002	959.50	< 0.00001
rs138928773	2	2	2	2	2	2	2	2	2	2	2	2	2	2	1	1	*[2]*	*2*	*2*	[1]	*[2]*	[1]	*[2]*	*[2]*	*[2]*	*[2]*	*[2]*	*[2]*	[1]	[1]	24/30	0.003	541.05	< 0.00001
rs528859858	2	2	2	2	2	2	2	2	2	2	2	2	2	2	2	1	*[2]*	*2*	*2*	[1]	*[2]*	[1]	*[2]*	*[2]*	*[2]*	*[2]*	*[2]*	*[2]*	[1]	[1]	25/30	0.002	363.15	< 0.00001
rs149218089	2	2	2	2	2	2	2	2	2	2	2	2	2	2	2	1	*2*	*2*	*2*	1	*2*	1	*2*	*2*	*2*	*2*	*2*	*2*	1	1	25/30	0.071	22.92	< 0.00001
rs555296478	2	2	2	2	2	2	2	2	2	2	2	2	2	2	2	2	*[2]*	*2*	*2*	*[2]*	*[2]*	*[2]*	*[2]*	*[2]*	*[2]*	*[2]*	*[2]*	*[2]*	*[2]*	*[2]*	30/30	0.003	731.18	< 0.00001
rs572272180	2	2	2	2	2	2	2	2	2	2	2	2	2	2	2	2	*[2]*	*2*	*2*	*[2]*	*[2]*	*[2]*	*[2]*	*[2]*	*[2]*	*[2]*	*[2]*	*[2]*	*[2]*	*[2]*	30/30	0.005	213.72	< 0.00001
rs115293800	2	2	2	2	2	2	2	2	2	2	2	2	2	2	2	2	*[2]*	*2*	*2*	*[2]*	*[2]*	*[2]*	*[2]*	*[2]*	*[2]*	*[2]*	*[2]*	*[2]*	*[2]*	*[2]*	30/30	0.024	165.23	< 0.00001
rs866411539	2	2	2	2	2	2	2	2	2	2	2	2	2	2	2	2	*[2]*	*2*	*2*	*[2]*	*[2]*	*[2]*	*[2]*	*[2]*	*[2]*	*[2]*	*[2]*	*[2]*	*[2]*	*[2]*	30/30	0.001	2887.48	< 0.00001
rs145962085	2	2	2	2	2	2	2	2	2	2	2	2	2	2	2	2	*[2]*	*2*	*2*	*[2]*	*[2]*	*[2]*	*[2]*	*[2]*	*[2]*	*[2]*	*[2]*	*[2]*	*[2]*	*[2]*	30/30	0.008	262.28	< 0.00001
rs1043184969	2	2	2	2	2	2	2	2	2	2	2	2	2	2	2	2	*[2]*	*2*	*2*	*[2]*	*[2]*	*[2]*	*[2]*	*[2]*	*[2]*	*[2]*	*[2]*	*[2]*	*[2]*	*[2]*	30/30	0.001	2744.98	< 0.00001
rs954450605	2	2	2	2	2	2	2	2	2	2	2	2	2	2	2	2	*[2]*	*2*	*2*	*[2]*	*[2]*	*2*	*[2]*	*2*	*2*	*2*	*2*	1	*2*	*[2]*	29/30	0.001	1411.43	< 0.00001
(ATTTC)n (Chr1:57,367,044-57,367,118)																																		
rs79992829	*[2]*	*[2]*	*[2]*	*[2]*	*[2]*	*[2]*	*[2]*	*[2]*	*[2]*	*[2]*	*[2]*	*[2]*	*[2]*	*[2]*	*[2]*	*[2]*	*[2]*	2	2	*[2]*	2	2	*[2]*	2	2	*[2]*	*[2]*	*[2]*	*[2]*	*[2]*	30/30	0.0092	3130.26	< 0.00001
rs146472695	*[2]*	*[2]*	*[2]*	*[2]*	*[2]*	*[2]*	*[2]*	*[2]*	*[2]*	*[2]*	*[2]*	*[2]*	*[2]*	*[2]*	*[2]*	*[2]*	*[2]*	2	2	*[2]*	2	2	*[2]*	2	2	*[2]*	*[2]*	*[2]*	*[2]*	*[2]*	30/30	0.0092	3134.61	< 0.00001
rs192485043	2	2	2	2	2	2	2	2	2	2	2	2	2	2	2	2	*[2]*	*2*	*2*	*[2]*	*[2]*	*2*	*[2]*	*[2]*	*[2]*	*[2]*	*2*	*[2]*	*[2]*	*[2]*	30/30	0.002	667,70	< 0.00001
rs145097803	2	2	2	2	2	2	2	2	2	2	2	2	2	2	2	2	*[2]*	2	2	*[2]*	2	2	*[2]*	2	2	*[2]*	*[2]*	*[2]*	*[2]*	*[2]*	30/30	0.005	433	< 0.00001
rs929412570	2	2	2	2	2	2	2	2	2	2	2	2	2	2	2	2	*[2]*	*2*	*2*	*[2]*	*[2]*	*2*	*[2]*	*2*	*2*	*2*	*2*	*2*	*2*	*[2]*	30/30	0.001	2866.86	< 0.00001
rs76443397	*[2]*	*[2]*	*[2]*	*[2]*	*[2]*	*[2]*	*[2]*	*[2]*	*[2]*	*[2]*	*[2]*	*[2]*	*[2]*	*[2]*	*[2]*	*[2]*	*2*	*2*	*2*	*2*	*[2]*	*2*	*2*	*2*	*2*	*2*	*2*	1	*2*	*2*	29/30	0.0064	4204.36	< 0.00001
rs151062149	[1]	[1]	[1]	[1]	[1]	[1]	[1]	[1]	[1]	[1]	[1]	[1]	[1]	[1]	[1]	[1]	1	1	2	[1]	[1]	1	[1]	1	1	1	1	1	1	1	1/30	0.0003	88.02	< 0.00001

Suppl. Table 17 Haplotype of SCA37 chromosomes in 30 patients from six Portuguese (PO) and eight Spanish (SP) kindreds included in the present study. Shaded haplotypes correspond to minor allele (2) SNP haplotypes shared by subsets of patients, which are altered by recombination or mutation events that lead to the presence of the reference allele (1). Furthermore, the framed haplotype indicates the 964 kb common haplotype common in studied individuals. Allelic phase inferred by PHASE software is shown in italics. In brackets are those alleles not genotyped but inferred by PHASE. Statistical significance of the Chi-square for the Linkage disequilibrium analysis is reported. MAF were obtained considering allele frequencies in Seixas *et al.* 2017 and the NCBI snp database (htttps://www.ncbi.nlm.nih.gov/snp/).

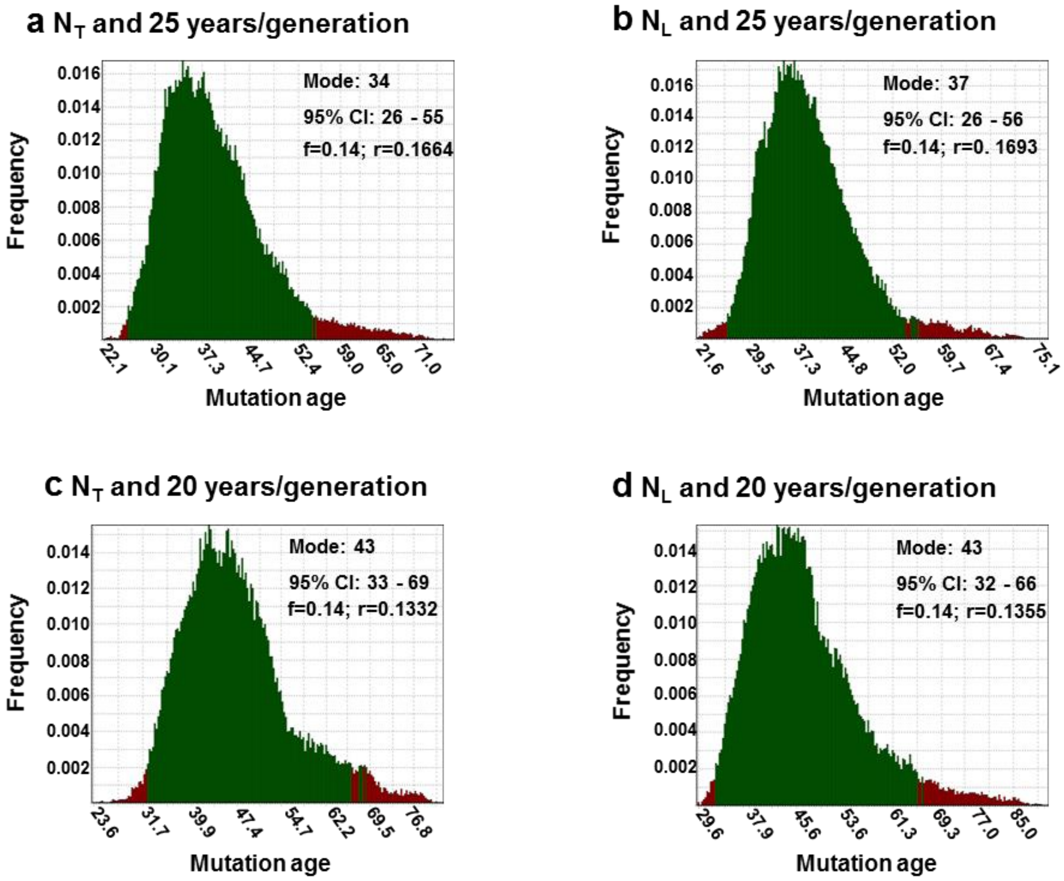

Suppl. Figure 11 Results from the DMLE+2.3. Analysis considering the total Iberian Peninsula population (N_T) or the southwest Iberian Peninsula (Huelva and Algarve) region (N_L) and 20 or 25 years/generation. The estimated time of the mutation is represented in generations ago with 95% CI being the 95% credible set of values.
